# Effect of Performance Improvement Programs on Compliance with Sepsis Bundles and Mortality: A Systematic Review and Meta-Analysis of Observational Studies

**DOI:** 10.1371/journal.pone.0125827

**Published:** 2015-05-06

**Authors:** Elisa Damiani, Abele Donati, Giulia Serafini, Laura Rinaldi, Erica Adrario, Paolo Pelaia, Stefano Busani, Massimo Girardis

**Affiliations:** 1 Anesthesia and Intensive Care Unit, Department of Biomedical Sciences and Public Health, Università Politecnica delle Marche, Via Tronto 10, 60126 Torrette di Ancona, Italy; 2 Department of Anesthesiology and Intensive Care, Modena University Hospital, L.go del Pozzo 71, 41100 Modena, Italy; University of Florida, UNITED STATES

## Abstract

**Background:**

Several reports suggest that implementation of the Surviving Sepsis Campaign (SSC) guidelines is associated with mortality reduction in sepsis. However, adherence to the guideline-based resuscitation and management sepsis bundles is still poor.

**Objective:**

To perform a systematic review of studies evaluating the impact of performance improvement programs on compliance with Surviving Sepsis Campaign (SSC) guideline-based bundles and/or mortality.

**Data Sources:**

Medline (PubMed), Scopus and Intercollegiate Studies Institute Web of Knowledge databases from 2004 (first publication of the SSC guidelines) to October 2014.

**Study Selection:**

Studies on adult patients with sepsis, severe sepsis or septic shock that evaluated changes in compliance to individual/combined bundle targets and/or mortality following the implementation of performance improvement programs. Interventions may consist of educational programs, process changes or both.

**Data Extraction:**

Data from the included studies were extracted independently by two authors. Unadjusted binary data were collected in order to calculate odds ratios (OR) for compliance to individual/combined bundle targets. Adjusted (if available) or unadjusted data of mortality were collected. Random-effects models were used for the data synthesis.

**Results:**

Fifty observational studies were selected. Despite high inconsistency across studies, performance improvement programs were associated with increased compliance with the complete 6-hour bundle (OR = 4.12 [95% confidence interval 2.95-5.76], *I^2^* = 87.72%, k = 25, N = 50,081) and the complete 24-hour bundle (OR = 2.57 [1.74-3.77], *I^2^* = 85.22%, k = 11, N = 45,846) and with a reduction in mortality (OR = 0.66 [0.61-0.72], *I^2^* = 87.93%, k = 48, N = 434,447). Funnel plots showed asymmetry.

**Conclusions:**

Performance improvement programs are associated with increased adherence to resuscitation and management sepsis bundles and with reduced mortality in patients with sepsis, severe sepsis or septic shock.

## Introduction

Sepsis is a major healthcare problem, with increasing incidence and persistently poor outcome [1]. The Surviving Sepsis Campaign (SSC) was first launched in 2002 with the goals of increasing clinician and public awareness of the sepsis problem, improving the standard of care and reducing mortality [2]. The SSC Guidelines for management of severe sepsis and septic shock were first published in 2004 and lastly updated in 2012 [3], providing recommendations that are intended to guide clinical practice. In order to facilitate the implementation of the guidelines, the key elements were organized into two bundles of care, the “resuscitation” and “management” bundle, including interventions to be accomplished within 6 and 24 hours, respectively [4]. A bundle is a set of diagnostic or therapeutic processes that when implemented as a group may act synergistically, providing a greater survival benefit than each individual component. Several reports showed that compliance with 6-hour and 24-hour sepsis bundles was associated with lower risk of death in patients with severe sepsis and septic shock [5, 6].

Nonetheless, transferring evidence into clinical practice has proven difficult. Adherence to SSC guidelines is still poor, especially among non-intensive care specialists [7]. Performance improvement initiatives (varying from educational programs [8], introduction of clinical decision support tools [9] or dedicated medical staff [10]) were instituted worldwide in the last years in order to address the piecemeal application of sepsis bundles. We performed a systematic review of studies that evaluated the impact of performance improvement programs on compliance to SSC guidelines and/or mortality in septic patients.

## Methods

This report adheres to the Preferred Reporting Items for Systematic reviews and Meta-Analysis (PRISMA) standards for reporting systematic review and meta-analysis studies (S1 Table). No review protocol exists for the present systematic review.

### Eligibility criteria

Studies were eligible for inclusion if they were observational (prospective or retrospective cohort or case-control studies) or randomized controlled trials (RCTs) investigating the effect of a performance improvement program on the implementation of sepsis care and/or patient outcome. Participants were required to be adult patients with sepsis, severe sepsis or septic shock. Sepsis had to be defined according to the criteria established by the American College of Chest Physicians and Society of Critical Care Medicine Consensus Conference [11]. The performance improvement program could be any intervention aimed at improving compliance to one or more components of the 6-hour or 24-hour sepsis bundles as based on the 2004 SSC guidelines [12] (Table 1). The comparator group included adult patients with sepsis, severe sepsis or septic shock who were treated before or without the influence of the implementation program. The primary outcome was changes in compliance to individual and/or combined bundle targets after the performance improvement program. The secondary outcome was mortality.

**Table 1:** Sepsis bundles.

**Resuscitation bundle (to be achieved within 6 hours from severe sepsis/septic shock diagnosis)**	
1 - Measure blood lactate	
2 - Blood cultures	*at least 2 sets of blood cultures before administration of antibiotics*
3 - Antibiotics	*broad-spectrum antibiotics within 3 hours of admission to the emergency department or within 1 hour of admission to other hospital units*
4 - SvO_2_	*measure and achieve central venous oxygen saturation >70%*
5 - Fluid resuscitation	*if hypotension and/or blood lactate >4 mmol/L*, *1 L crystalloids (or 0*.*5 L of colloid equivalent) in 30 minutes*
6 - Central Venous Pressure	*if hypotension despite fluid resuscitation and/or blood lactate >4 mmol/L*, *achieve CVP >8 mmHg*
7 - Vasopressors	*if hypotension not responding to fluid resuscitation*, *maintain a mean arterial pressure >65 mmHg*
**Management bundle (to be achieved within 24 hours from severe sepsis diagnosis)**	
1 - Lung protective ventilation	*maintain inspiratory plateau pressures <30 cmH*_*2*_ *O for mechanically ventilated patients; avoid a tidal volume >6 mL/kg for patients with acute respiratory distress syndrome*
2 - Steroids	*administer low-dose steroids for septic shock in accordance with a standardized hospital policy*
3 - Drotrecogin alfa (activated)	*in accordance with a standardized hospital policy*
4 - Glucose control	*> 4 mmol/L but <8*.*3 mmol/L*

### Search strategy

Medline (PubMed), Scopus and Intercollegiate Studies Institute (ISI) Web of Knowledge databases were searched from 2004 (first publication of the SSC guidelines). The main search was run on 29th May 2014 and updated weekly until October 2014. The keywords “sepsis”, “septic shock”, “bundle”, “bundled care”, “guidelines”, “surviving sepsis campaign”, “implementation”, “compliance”, “performance improvement program”, “quality improvement program” were typed in various combinations using boolean operators (see the detailed search strategy in S1 Text). Hand searches of reference lists of articles and relevant literature reviews were used to complement the computer search. Content pages of the main journals on critical care medicine were hand-searched in order to find any relevant in-press article. The search was limited to English language studies published in peer-reviewed journals.

### Study selection and data extraction

Two independent reviewers screened all identified records (title and abstract) and assessed the selected full-text articles for eligibility. Disagreements were resolved through discussion. Descriptive, methodological and outcome data were extracted from all the eligible studies by two reviewers who worked independently using a predefined data extraction form. The following data were collected: design, study period, country, number of centers included, setting (emergency department [ED], intensive care unit [ICU], hospital), type of center (teaching/non-teaching hospital), type of admission (medical, surgical, mixed), mean age, gender distribution, severity of sepsis (sepsis, severe sepsis and/or septic shock), severity scores (Acute Physiology and Chronic Health Evaluation [APACHE] II score if available; alternatively the Sequential Organ Failure Assessment [SOFA] or the Simplified Acute Physiology Score [SAPS] II), characteristics of the performance improvement program implemented, data for primary and secondary outcome of interest. Severity of illness was recoded as a dichotomous variable based on an expected mortality <50% (APACHE II≤23 or SAPS II≤51 or SOFA≤12, “low severity”) or ≥50% (APACHE II≥24 or SAPS II≥52 or SOFA≥13, “high severity”). Performance improvement programs were categorized as educational only, process change only or both educational and process change based on the type of interventions implemented. It was defined as educational any program aimed at increasing the awareness of sepsis disease by educating medical/nurse staff in sepsis pathogenesis, diagnosis and treatment and promoting the widespread divulgation of this information. An educational program may consist of lectures and training sessions, posters, pocket/bedside reminder cards. A program was categorized as process change if it was intended to induce variations in the standard sepsis management by means of clinical decision support tools (screening tools, predefined order sets, treatment algorithms as flow-charts or checklists, computer applications for bedside monitoring and alert systems, laboratory routine exam sets for sepsis) or the introduction of dedicated staff (sepsis teams, sepsis dedicated units).

### Assessment of risk of bias and study quality

Risk of bias was assessed by determining whether each study controlled for two pre-defined confounding factors: illness severity (as defined by APACHE II, SOFA or SAPS) and age. These factors were selected as expected to exert the greatest impact on the outcome, besides being the ones most widely reported by the authors. A study was considered to adequately control for each of these factors if it: *a*—restricted participant selection so that both groups had the same value for the confounder; *b*—demonstrated balance between groups for the confounder (p>0.05 in the between-group comparison and/or absolute difference ≤10%); *c*—matched on the confounder; or *d*—adjusted for the confounder in statistical analyses to quantify the effect size (ES) for mortality and reported adjusted results. Study quality was assessed by means of the Newcastle-Ottawa Scale (NOS) for cohort/case-control studies [13]. Groups (cohorts/cases and controls) were considered to be comparable if the study controlled for illness severity and/or age. The item was considered as non-fulfilled if the authors adjusted for illness severity and/or age but results of the multivariable models were not reported. Adequacy of follow-up was considered as fulfilled if at least hospital or 28-day mortality were reported. Completeness of follow-up was fulfilled if the authors reported a number/percentage of patients excluded from the analysis because of missing mortality data ≤10% of total. If the number of exclusions was not stated clearly, the item was considered as non-fulfilled.

### Statistical analysis

Odds ratio (OR) was chosen as the effect estimate for the data synthesis. As the majority of studies did not report adjusted ORs for the primary outcome of interest (compliance to individual SSC guidelines targets or bundles), unadjusted binary data (number if available, otherwise percentage of patients in which the item was fulfilled in intervention and control groups) were extracted for the calculation of ORs. For the secondary outcome (mortality), adjusted ORs were extracted whenever available; if the authors reported the results of more than one multivariable regression models, the one including severity of illness (as represented by APACHE II, SOFA or SAPS) and/or age was selected; if adjusted ORs were not reported or if the authors reported different effect estimates (Hazard Ratio [HR], Relative Risk [RR]), unadjusted binary data were extracted and used for the reconstruction of OR and data synthesis. Adjusted HR or RR values were not used in the statistical analysis. The influence of this method of analysis (use of adjusted or unadjusted data) on the calculated ES was tested through sensitivity analysis. Hospital mortality was considered whenever available, otherwise the longest-term mortality was used. Data were synthesized using meta-analytic methods [14, 15], and statistically pooled by the standard meta-analysis approach, i.e. studies were weighted by the inverse of the sampling variance. The DerSimonian and Laird random effects model was used as a conservative approach to account for different sources of variation among studies. Forest plots were constructed to graphically represent the results. Q statistics were used to assess heterogeneity among studies. A significant Q value indicates a lack of homogeneity of findings among studies [14, 15]. *I* ^*2*^ statistics were then used to quantify the proportion of observed inconsistency across study results not explained by chance [16]. *I* ^*2*^ values of <25%, 50% and >75% represent low, moderate and high inconsistency, respectively [16]. Several variables were identified and their effects on outcome examined. Sensitivity analyses were performed by excluding each single study at a time in order to assess the influence of each study on the pooled ES. Categorical variables were treated as moderators and the ES was assessed and compared across subgroups formed by these moderators. Continuous variables were examined as covariates using random effects meta-regression. Meta-regression and sub-group analyses were performed to assess the effect of study quality (NOS score, risk of bias) on the calculated estimates. The presence of publication bias was investigated through funnel plots both visually and formally by trim and fill analysis and Eggers’s linear regression method [17]. A p value less than 0.05 was used to indicate statistical significance. All analyses were conducted using a computer software package (ProMeta Version 2, Internovi, Cesena FC, Italy).

## Results

**Fig 1:**
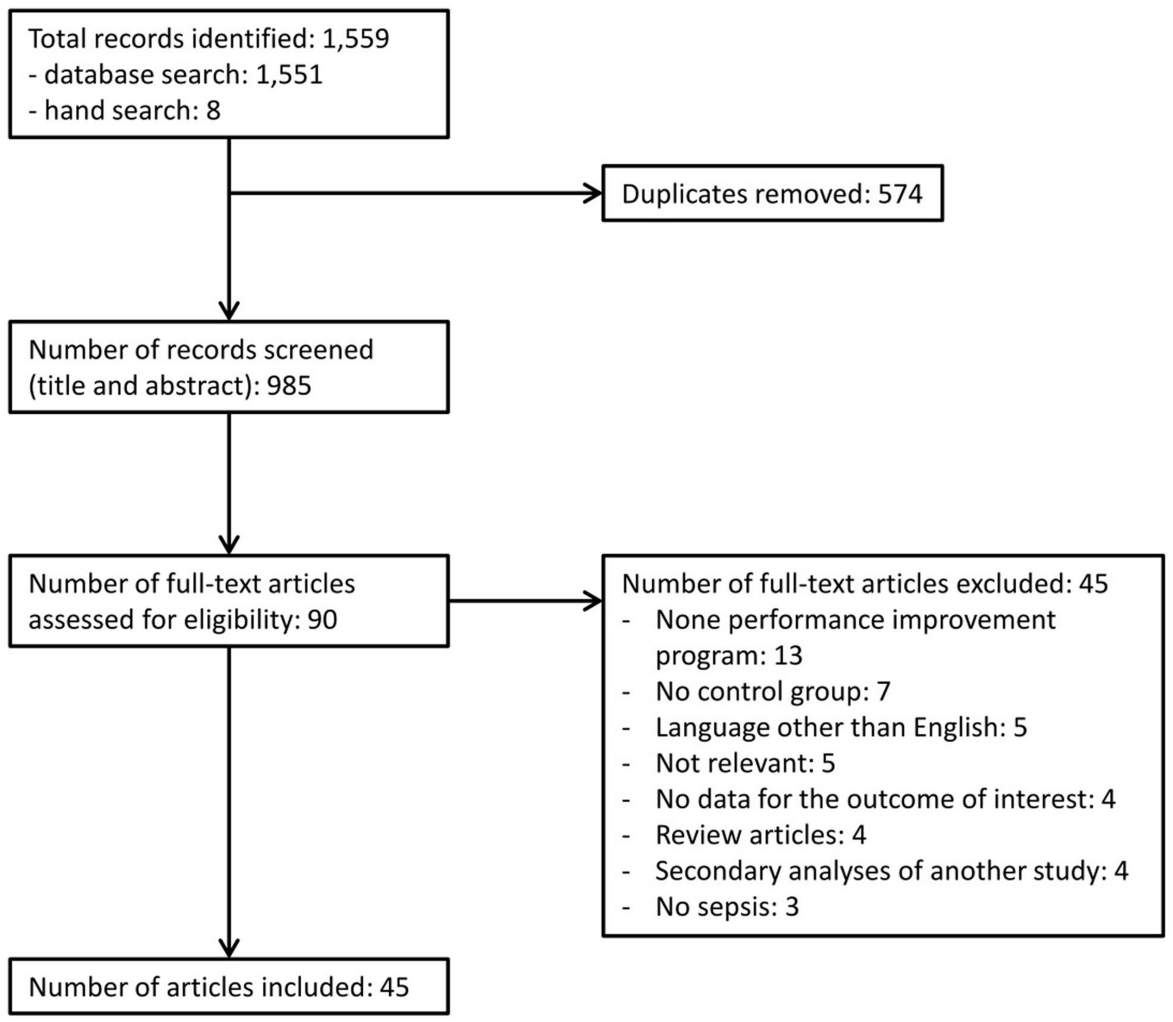
Flow-chart showing the study selection process.

The study selection process is described in Fig 1. Among the initial 1,560 records, 50 studies published between 2006 and 2014 met our inclusion criteria [6, 8–10, 18–63]. Study characteristics are shown in S2 Table. Three studies evaluated two interventions in subsequent periods [53, 57, 60]; each part of these studies was included separately in order to consider the effect of each different intervention. Of the 53 resulting studies, the majority was prospective (23 before-after, 5 time-series analyses, 2 cohort, 1 case-control), 11 were retrospective (8 before-after, 2 cohort, 1 case-control), 11 were historically controlled investigations. No eligible RCT was found. Thirty-eight studies were single-center (72%), 15 were multi-center (28%). Thirty-six studies (68%) out of 53 were on patients with severe sepsis or septic shock, 11 (21%) also included patients with sepsis, 6 (11%) focused only on patients with septic shock. The performance improvement programs implemented were only educational in 17 studies out of 53 (32%), only process changes in 13 (25%), both educational and process changes in 23 (43%). A more detailed description of each program is provided in S2 Table. These programs were implemented in ICU (22 studies; 41%), ED (18; 34%), or the whole hospital (13; 25%). The participating centers were teaching hospitals in most cases (31 studies; 58%). In eight studies the post-intervention phase had been divided in different time periods in order to observe the effect of the performance improvement programs over time (8, 20, 27, 38, 43, 46, 48, 54); in these cases, the final post-intervention period was considered for our analysis. Outcomes measured in each individual study are shown in S3 Table. Risk of bias and study quality assessment is reported in S4 Table.

### Compliance with 6-hour sepsis bundle

**Fig 2:**
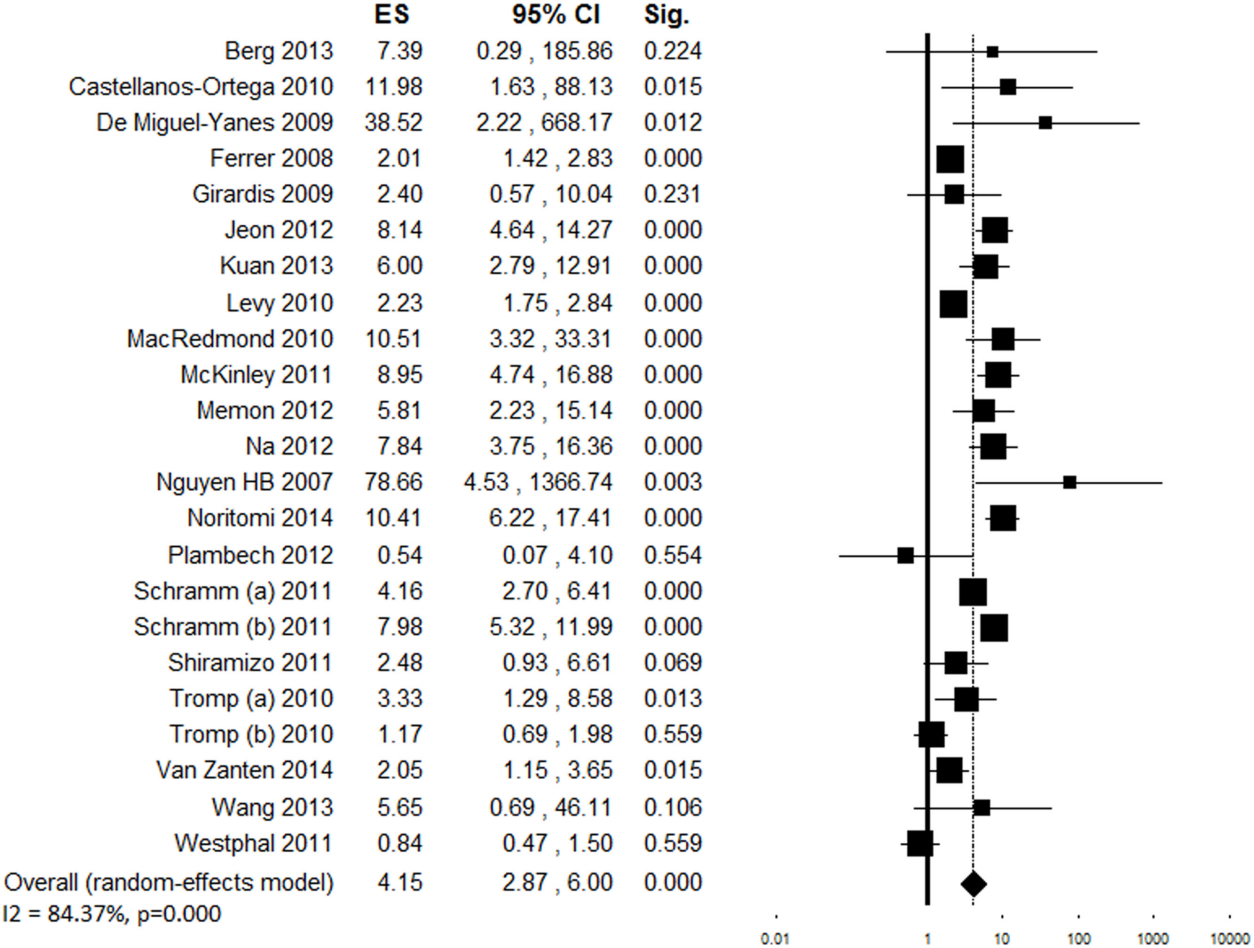
Forest plot showing individual and overall ES of studies that evaluated changes in compliance with the complete 6-hour bundle following the implementation of the performance improvement program (k = 25). The size of the boxes is inversely proportional to the size of the result study variance, so that more precise studies have larger boxes. The ES is expressed as odds ratio (OR) and the correspondent 95% confidence interval (CI). An OR above 1.00 (right side of the plot) indicates an association between the intervention and increased compliance. ES = effect size; CI = confidence interval; Sig. = p value.

Twenty-five studies reported changes in compliance with the complete 6-hour bundle after the performance improvement program, for a total of 50,081 episodes of sepsis, severe sepsis or septic shock evaluated [6, 8, 10, 21, 23, 25, 27, 32, 34, 38, 39–41, 45, 46, 48, 51, 53, 54, 56, 60, 62, 63]. Despite high inconsistency between studies (*Q* (24) = 195.48, p <0.001, *I* ^*2*^ = 87.72%), the majority showed a significant increase in compliance (Fig 2). The overall ES indicated a positive association between the quality improvement interventions and compliance with the resuscitation bundle (OR = 4.12 [95% confidence interval 2.95–5.76], p <0.001, Fig 2). This result did not change significantly when any of the included studies was removed from the analysis in the sensitivity analysis. Funnel plot showed asymmetry (S1 Fig), however with a minor effect on the ES as indicated by the trim and fill analysis (estimated ES: OR = 3.45 [2.49–4.78], p <0.001; number of trimmed studies: 5). The Egger’s linear regression test confirmed the possible presence of a publication bias (p = 0.005). We searched for possible sources of heterogeneity related to different characteristics of studies, patients or programs implemented (Table 2). The ES significantly changed based on the study design (lower ES shown by time-series analyses) and region (highest ESs for studies conducted in North America and Asia). Seven multi-center studies showed lower ES as compared to single-center investigations (k = 18). Studies on patients with higher illness severity (k = 5) consistently showed a larger increase in compliance as compared to those on patients with a lower risk of death. Ten studies that implemented both education and process change programs yielded a higher increase in compliance as compared to those implementing educational (k = 10) or process change (k = 5) interventions alone. A consistent improvement was observed among studies with a lower initial compliance. A greater increase in compliance was observed among studies where only teaching hospitals had been involved (k = 13) as compared to those where both teaching and non-teaching centers had participated (k = 5).

**Table 2:** Moderator analysis: compliance with the complete 6-hour sepsis bundle (k = 25).

	*k*	*N*	ES	95% CI	p	Q	I^2^	p^a^
***Design***								<0.001
Prospective before-after	13	6225	3.71	2.16–6.36	<0.001	105.00***	88.57	
Historically controlled	5	1321	8.31	5.40–12.79	<0.001	1.94	0.00	
Time-series analysis	5	42295	2.22	1.56–3.15	<0.001	14.02**	71.46	
Prospective cohort	1	117	6.00	2.79–12.91	<0.001	-	-	
Retrospective cohort	1	123	8.39	0.29–185.86	0.224	-	-	
***Number of centers***								0.019
Single-center	18	5841	5.47	3.50–8.54	<0.001	65.80***	74.16	
Multi-center	7	44240	2.64	1.75–3.99	<0.001	62.69***	90.43	
***Region***								<0.001
Europe	8	12516	2.11	1.36–3.26	0.001	13.86	49.49	
North America	7	2838	8.85	4.99–15.68	<0.001	16.64*	63.94	
Asia	5	1159	7.15	5.02–10.17	<0.001	0.69	0.00	
South America	3	798	2.81	0.51–15.55	0.237	4.85***	95.10	
***Sepsis severity***								0.013
Sepsis + severe sepsis + septic shock	5	1514	1.53	0.72–3.27	0.267	12.14*	67.05	
Severe sepsis + septic shock	19	48087	4.98	3.44–7.20	<0.001	165.23***	89.11	
Septic shock	1	480	11.98	1.63–88.13	0.015	-	-	
***Severity of illness***								0.047
Low	13	13904	4.61	2.61–8.14	<0.001	83.34***	85.60	
High	5	961	10.08	6.00–16.95	<0.001	2.19	0.00	
***Type of admission***								0.003
Mixed	12	45282	2.86	2.00–4.10	<0.001	65.98***	83.33	
Medical	3	2357	8.24	3.25–20.85	<0.001	12.09**	83.46	
Surgical	1	206	8.95	4.74–16.88	<0.001	-	-	
***Baseline compliance (6h bundle)***								0.444
<10%	7	4114	2.92	1.76–4.85	<0.001	9.99	39.94	
10–25%	10	35715	4.57	2.85–7.30	<0.001	135.09***	93.34	
>25%	5	9948	4.09	1.23–13.59	0.021	40.71***	90.17	
***Type of intervention***								0.027
Education	10	45281	2.91	2.07–4.09	<0.001	57.81***	84.43	
Process change	5	1271	3.52	1.00–12.39	0.049	30.29***	86.79	
Education + Process change	10	3529	6.56	4.03–10.68	<0.001	22.93**	60.75	
***Setting***								0.550
Emergency Department	9	1944	4.95	2.19–11.19	<0.001	37.26***	78.53	
Intensive Care Unit	10	14297	4.61	2.75–7.74	<0.001	49.96***	81.99	
Hospital	6	33840	3.14	1.75–5.64	<0.001	73.35***	93.18	
***Type of center***								0.005
Teaching hospital	13	4153	5.70	3.52–9.22	<0.001	50.83***	76.39	
Non-teaching hospital	5	1960	4.84	1.21–19.36	0.026	26.29***	84.78	
Mixed	5	43476	2.31	1.76–3.03	<0.001	15.07**	73.46	

*p<0.05

**p<0.01

***p<0.001

^a^ p for comparison between subgroups

**Fig 3:**
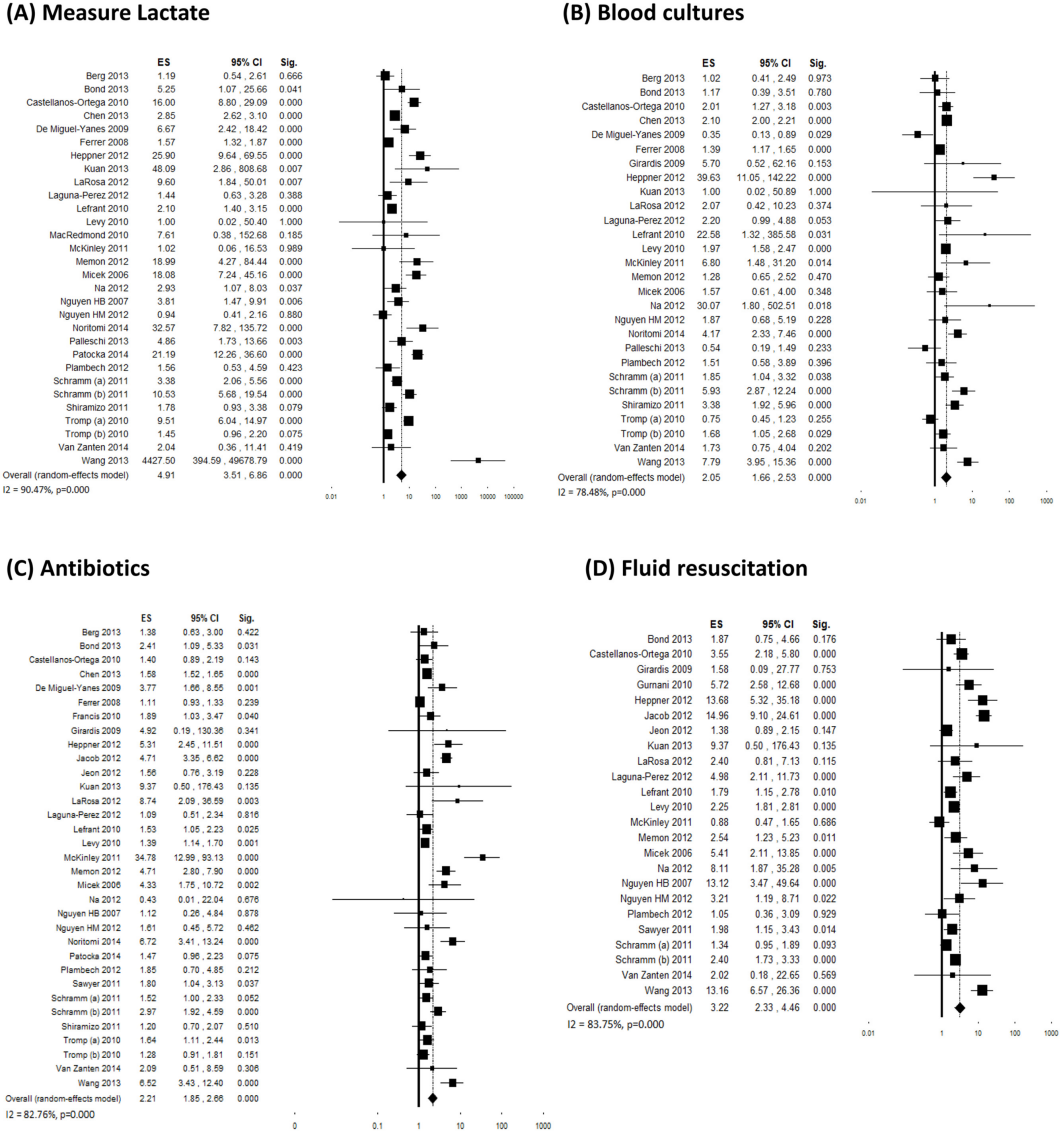
Forest plots showing individual and overall ES of studies that evaluated changes in compliance with individual 6-hour bundle targets following the implementation of the performance improvement program. (A) Measure lactate (k = 31); (B) Blood cultures (k = 28); (C) Antibiotics (k = 35); (D) Fluid resuscitation (k = 24). The size of the boxes is inversely proportional to the size of the result study variance, so that more precise studies have larger boxes. The ES is expressed as odds ratio (OR) and the correspondent 95% confidence interval (CI). An OR above 1.00 (right side of the plot) indicates an association between the intervention and increased compliance. ES = effect size; CI = confidence interval; Sig. = p value.

**Fig 4:**
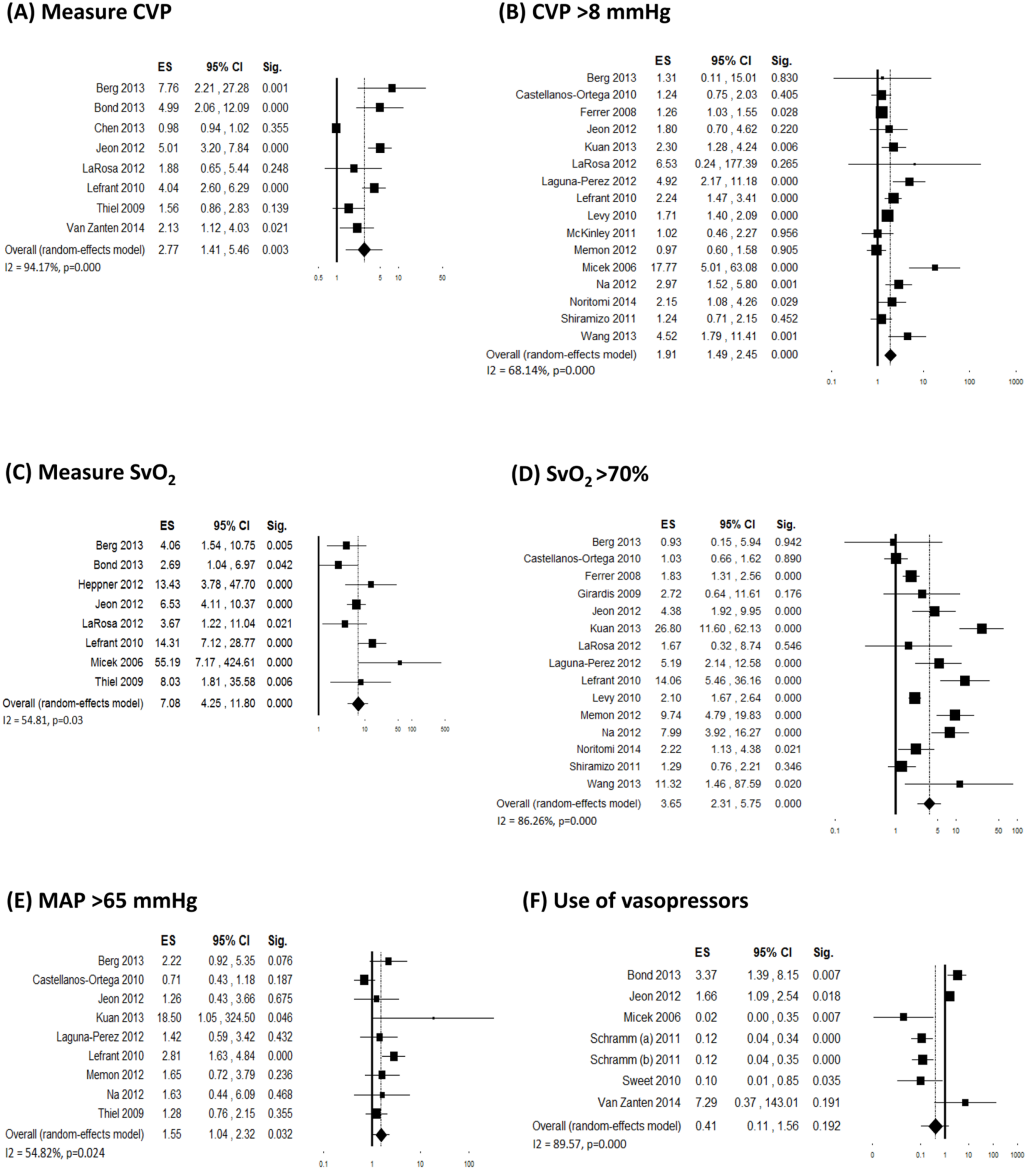
Forest plots showing individual and overall ES of studies that evaluated changes in compliance with individual 6-hour bundle targets following the implementation of the performance improvement program. (A) Measure central venous pressure (k = 8); (B) Central venous pressure above 8 mmHg (k = 16); (C) Measure SvO_2_ (k = 8); (D) SvO_2_ above 70% (k = 15); (E) Mean arterial pressure above 65 mmHg (k = 9); (F) Use of vasopressors (k = 9). The size of the boxes is inversely proportional to the size of the result study variance, so that more precise studies have larger boxes. The ES is expressed as odds ratio (OR) and the correspondent 95% confidence interval (CI). An OR above 1.00 (right side of the plot) indicates an association between the intervention and increased compliance. ES = effect size; CI = confidence interval; Sig. = p value.

A variable number of studies (ranging from 8 to 35) evaluated changes in compliance with individual 6-hour bundle targets. Individual ES and overall estimates are graphically shown in Figs 3 and 4. Although inconsistency between studies was generally high, an increased adherence was visible for all bundle components. Four studies [42, 53, 58] demonstrated a significant decrease in the use of vasopressors after the interventions, which was probably a consequence of the increased adherence to fluid resuscitation. The majority of the funnel plots showed asymmetry, however with a minor effect on the final ES in most cases (S2 Fig).

### Compliance with 24-hour sepsis bundle

**Fig 5:**
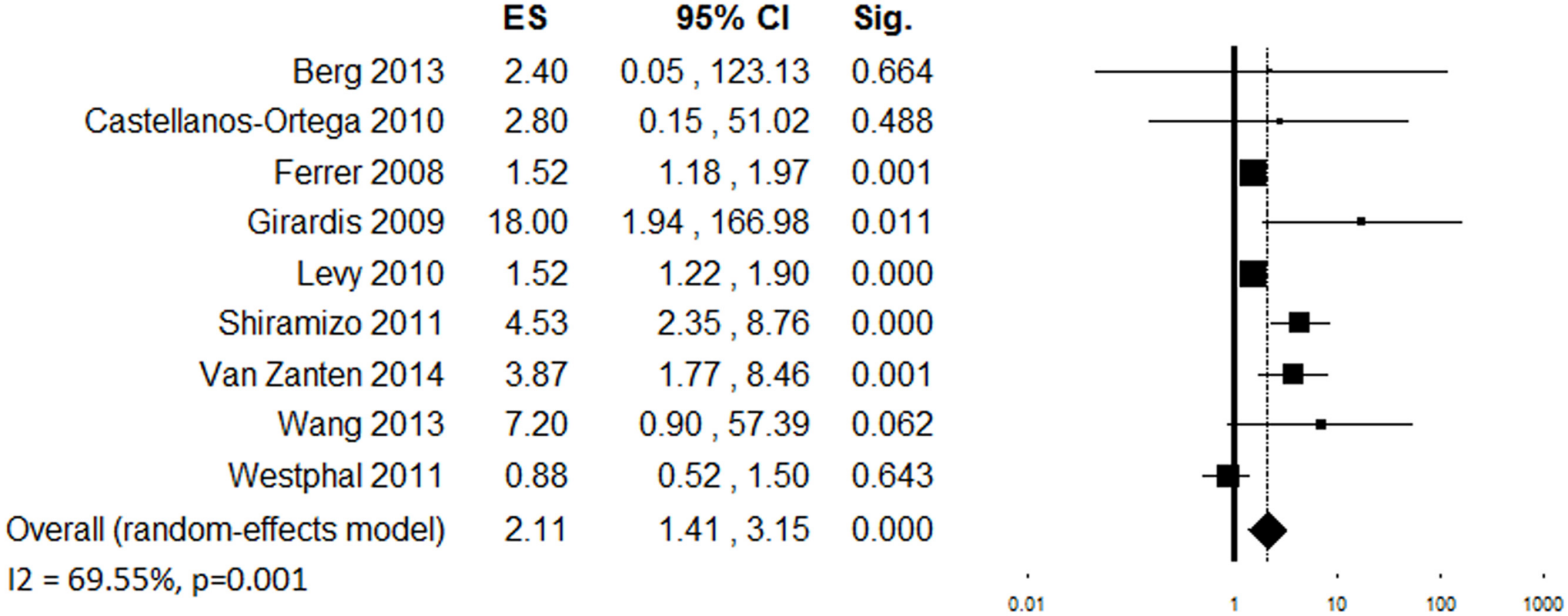
Forest plot showing individual and overall ES of studies that evaluated changes in compliance with the complete 24-hour bundle following the implementation of the performance improvement program (k = 11). The size of the boxes is inversely proportional to the size of the result study variance, so that more precise studies have larger boxes. The ES is expressed as odds ratio (OR) and the correspondent 95% confidence interval (CI). An OR above 1.00 (right side of the plot) indicates an association between the intervention and increased compliance. ES = effect size; CI = confidence interval; Sig. = p value.

Eleven studies evaluated changes in compliance with the complete 24-hour bundle following the performance improvement program [6, 8, 10, 21, 25, 27, 38, 54, 56, 62, 63] in a total of 45,846 patients. Most of the studies reported a significant increase in compliance following the implementation of the program, although inconsistency between study results was high (*Q* (10) = 67.97, p<0.001, *I* ^*2*^ = 85.22%). The combined OR was 2.57 [1.74–3.77] (p<0.001) (Fig 5). Sensitivity analysis showed that the removal of any of the included studies did not change the overall OR significantly. The funnel plot showed asymmetry (S3 Fig). The trim and fill analysis revealed a possible significant effect of the publication bias on the overall ES (estimated ES: OR = 1.43 [0.94–2.17] p = 0.094, number of trimmed studies: 5). The Egger’s linear regression test confirmed the possibility of a publication bias (p = 0.048). In the subgroup analyses (Table 3), single-center studies (k = 6) showed higher increase in the adherence to 24-hour bundle in comparison to multi-center studies (k = 5). Four studies that implemented both educational and process change programs showed higher increase in compliance with 24-hour bundle as compared to studies in which only educational or process change interventions were implemented; pure process change programs did not produce a significant improvement in compliance (k = 3). A larger and consistent effect was observed among studies that reported the lowest compliance at baseline (k = 2). A greater increase in compliance tended to be shown by studies where only teaching hospitals had been involved (k = 3) as compared to those where both teaching and non-teaching centers had participated (k = 4), while studies involving only non-teaching hospital (k = 4) did not show a significant improvement in the adherence to the 24-hour bundle.

**Table 3:** Moderator analysis: compliance with 24-hour sepsis bundle (k = 11).

	*k*	*N*	ES	95% CI	p	Q	I^2^	p^a^
***Design***								0.671
Prospective before-after	4	2948	2.00	0.98–4.08	0.056	16.54**	81.87	
Historically controlled	1	480	2.80	0.15–51.02	0.488	-	-	
Time-series analysis	5	42295	3.63	1.96–6.72	<0.001	50.86***	92.14	
Retrospective cohort	1	123	2.40	0.05–123.13	0.664	-	-	
***Number of centers***								0.003
Single-center	6	2153	9.69	2.91–32.30	<0.001	15.39**	67.52	
Multi-center	5	43693	1.49	1.23–1.81	<0.001	9.60*	58.31	
***Region***								0.451
Europe	4	11219	3.00	1.20–7.50	0.018	9.36*	67.93	
North America	2	1228	20.36	1.31–317.53	0.032	2.15	53.52	
Asia	1	195	7.20	0.90–57.39	0.062	-	-	
South America	2	434	1.98	0.40–9.83	0.406	14.33***	93.02	
***Sepsis severity***								0.002
Sepsis + severe sepsis + septic shock	1	217	0.88	0.52–1.50	0.643	-	-	
Severe sepsis + septic shock	9	45149	3.04	1.99–4.64	<0.001	63.02***	87.31	
Septic shock	1	480	2.80	0.15–51.02	0.488	-	-	
***Severity of illness***								0.674
Low	7	12473	4.53	1.89–10.85	0.001	61.40***	90.23	
High	2	603	2.65	0.26–27.43	0.414	0.00	0.00	
***Baseline compliance (24h bundle)***								0.032
<10%	2	228	11.02	2.41–50.33	0.002	0.35	0.00	
10–25%	4	35306	1.66	1.32–2.10	<0.001	11.05*	72.84	
>25%	3	9709	5.34	0.64–44.73	0.123	45.46***	95.60	
***Type of intervention***								0.044
Education	4	43476	1.56	1.32–1.84	<0.001	5.90	49.11	
Process change	3	535	1.80	0.41–7.91	0.436	3.87	48.32	
Education + Process change	4	1835	11.58	2.42–55.48	0.002	14.96**	79.95	
***Setting***								0.012
Emergency Department	1	195	7.20	0.90–57.39	0.062	-	-	
Intensive Care Unit	6	12541	6.03	2.04–17.80	0.001	50.15***	90.03	
Hospital	4	33110	1.43	1.25–1.64	<0.001	3.61	16.93	
***Type of center***								0.027
Teaching hospital	3	708	8.21	2.14–31.35	0.002	1.02	0.00	
Non-teaching hospital	4	757	5.01	0.79–31.75	0.087	47.45***	93.68	
Mixed	4	43476	1.56	1.32–1.84	<0.001	5.90	49.11	

*p<0.05

**p<0.01

***p<0.001

^a^ p for comparison between subgroups

**Fig 6:**
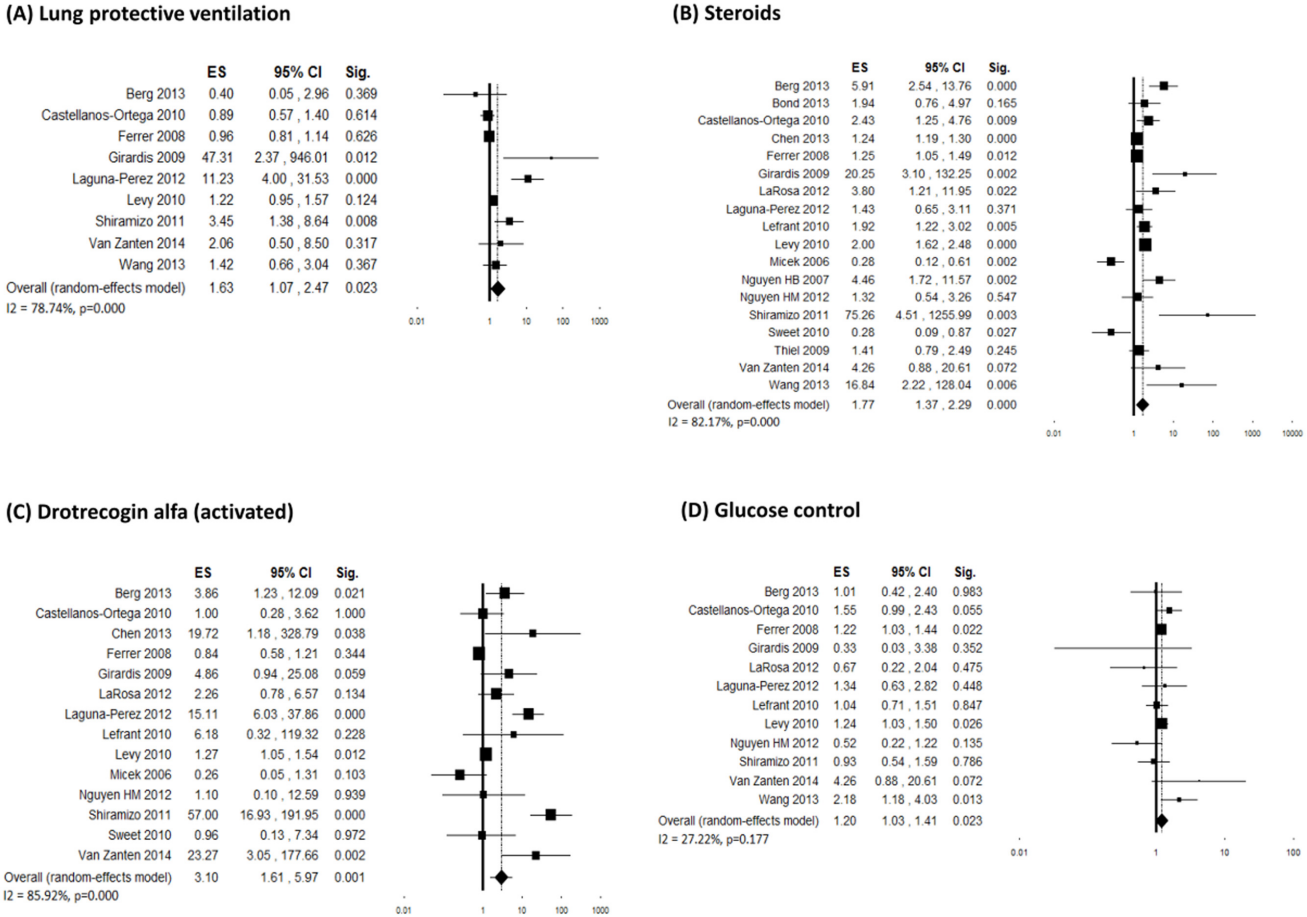
Forest plots showing individual and overall ES of studies that evaluated changes in compliance with individual 24-hour bundle targets following the implementation of the performance improvement program. (A) Lung protective ventilation (k = 9); (B) Steroids (k = 20); (C) Drotrecogin alfa (activated) (k = 16); (D) Glucose control (k = 13). The size of the boxes is inversely proportional to the size of the result study variance, so that more precise studies have larger boxes. The ES is expressed as odds ratio (OR) and the correspondent 95% confidence interval (CI). An OR above 1.00 (right side of the plot) indicates an association between the intervention and increased compliance. ES = effect size; CI = confidence interval; Sig. = p value.

A variable number of studies evaluated changes in compliance with individual 24-hour bundle targets (lung protective ventilation: 9; low-dose steroids: 20; Drotrecogin alfa activated: 16; glucose control: 13). High inconsistency across study findings was observed in general. Individual ES, overall estimates and heterogeneity analysis are shown in Fig 6. Funnel plots for the outcomes lung-protective ventilation and low-dose steroids showed asymmetry (S4 Fig).

### Mortality

**Fig 7:**
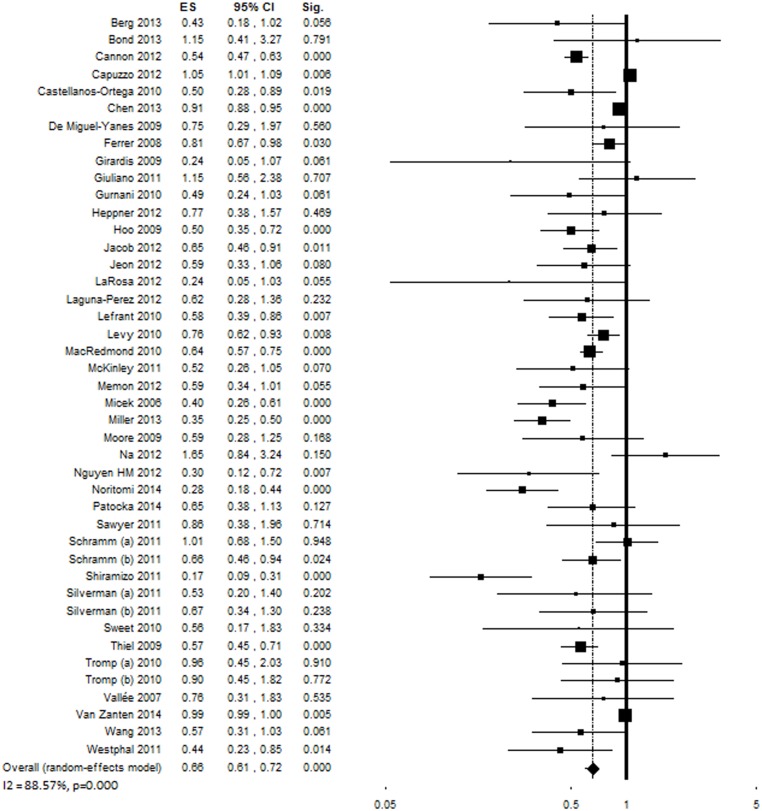
Forest plot showing individual and overall ES of studies that evaluated changes in mortality following the implementation of the performance improvement program (k = 48). The size of the boxes is inversely proportional to the size of the result study variance, so that more precise studies have larger boxes. The ES is expressed as odds ratio (OR) and the correspondent 95% confidence interval (CI). An OR below 1.00 (left side of the plot) indicates an association between the intervention and decreased mortality. ES = effect size; CI = confidence interval; Sig. = p value.

Forty-eight studies evaluated changes in mortality following the implementation of the performance improvement program [6, 8–10, 18–25, 27–33, 35–45, 47, 48, 50, 52–63] in a total of 434,447 patients. Despite high inconsistency between study findings (*Q* (47) = 389.39, p<0.001, *I* ^*2*^ = 87.93%), the majority of studies showed a significant decrease in mortality; the overall OR was 0.66 [0.61–0.72], p<0.001 (Fig 7). The removal of any of the included studies in the sensitivity analysis did not change the results significantly. Funnel plot analysis indicated a possible publication bias (S5 Fig), however with a minor effect on the final ES (estimated ES: OR = 0.77 [0.71–0.83], p<0.001; number of trimmed studies: 15). The Egger’s linear regression test confirmed the possible presence of a publication bias (p<0.001). Subgroup analyses were performed in order to investigate potential sources of heterogeneity (Table 4). The association between performance improvement program and mortality was influenced by study design (with time-series analyses showing higher ES as compared to historically controlled or before-after investigations), number of centers included (stronger association with reduced mortality among single-center studies), and region. The ES was influenced by the type of intervention. The strongest association with reduced mortality was shown by the studies that implemented both educational and process change programs (k = 22).

**Table 4:** Moderator analysis of studies that evaluated changes in mortality following the performance improvement program (k = 48).

	*k*	*N*	ES	95% CI	p	Q	I^2^	p^a^
***Design***								0.003
Prospective before-after	21	341562	0.63	0.50–0.78	<0.001	136.14***	85.31	
Historically controlled	11	7416	0.60	0.54–0.65	<0.001	5.12	0.00	
Retrospective before-after	6	41331	0.64	0.47–0.88	0.006	28.44***	82.42	
Time-series analysis	5	42295	0.80	0.66–0.98	0.033	28.58***	86.00	
Retrospective cohort	2	1413	0.36	0.26–0.50	<0.001	0.17	0.00	
Prospective cohort	1	270	0.86	0.38–1.96	0.714	-	-	
Prospective case-control	1	58	0.24	0.05–1.03	0.055	-	-	
Retrospective case-control	1	102	1.15	0.41–3.27	0.791	-	-	
***Number of centers***								0.001
Single-center	35	9598	0.59	0.53–0.67	<0.001	48.58	30.01	
Multi-center	13	424849	0.76	0.69–0.83	<0.001	200.85***	94.03	
***Region***								<0.001
North America	25	12685	0.58	0.51–0.65	<0.001	36.90*	34.97	
Europe	12	346857	0.95	0.88–1.02	0.161	32.96**	66.62	
Asia	5	40748	0.79	0.58–1.08	0.142	9.91*	59.63	
South America	3	798	0.27	0.17–0.45	<0.001	4.50	55.58	
Africa	1	616	0.65	0.46–0.91	0.011	-	-	
***Sepsis severity***								0.206
Sepsis + severe sepsis + septic shock	9	2851	0.67	0.53–0.84	<0.001	8.29	3.50	
Severe sepsis + septic shock	33	430494	0.68	0.62–0.74	<0.001	340.51***	90.60	
Septic shock	6	1102	0.53	0.41–0.69	<0.001	5.00	0.00	
***Severity of illness***								0.573
Low	18	15440	0.57	0.45–0.73	<0.001	144.92***	88.27	
High	11	1361	0.62	0.55–0.70	<0.001	6.19	0.00	
***Type of admission***								0.678
Mixed	20	425546	0.69	0.62–0.75	<0.001	298.83***	93.64	
Medical	6	2856	0.72	0.53–0.96	0.027	7.29	31.37	
Surgical	4	801	0.58	0.40–0.85	0.005	0.30	0.00	
***Baseline compliance with 6h bundle***								0.452
<10%	6	4115	0.54	0.34–0.84	0.007	25.20***	80.16	
10–25%	9	35652	0.72	0.59–0.87	0.001	35.03***	77.16	
>25%	6	10081	0.60	0.36–0.97	0.039	19.82**	74.77	
***Baseline compliance with 24h bundle***								0.710
<10%	2	228	0.49	0.26–0.93	0.018	1.11	10.09	
10–25%	4	35306	0.65	0.48–0.87	0.004	24.38***	87.69	
>25%	4	9842	0.67	0.38–1.19	0.172	13.14**	77.16	
***Type of intervention***								<0.001
Education	14	420303	0.86	0.79–0.92	<0.001	101.67***	87.21	
Process change	12	2938	0.66	0.55–0.80	<0.001	7.96	0.00	
Education + Process change	22	11206	0.53	0.46–0.62	<0.001	48.76**	56.93	
***Setting***								0.742
Emergency Department	13	2974	0.65	0.53–0.81	<0.001	18.72	35.90	
Intensive Care Unit	22	56041	0.69	0.61–0.77	<0.001	133.81***	84.31	
Hospital	13	374527	0.63	0.50–0.78	<0.001	175.10***	93.15	
***Type of center***								<0.001
Teaching hospital	27	6599	0.63	0.58–0.69	<0.001	26.67	2.51	
Non-teaching hospital	10	2927	0.48	0.37–0.63	<0.001	18.12*	50.33	
Mixed	10	423652	0.81	0.74–0.89	<0.001	158.12***	94.31	

*p<0.05

**p<0.01

***p<0.001

^a^ p for comparison between subgroups

### Sensitivity analysis

In meta-regression analyses, study quality (as indicated by the NOS score) did not show any significant impact on the observed ESs (p = 0.809 for compliance with 6-hour bundle; p = 0.397 for compliance with 24-hour bundle; p = 0.705 for mortality). Sensitivity analyses for primary and secondary outcomes were performed by excluding the studies that did not control for confounders (severity of illness and age) and had an NOS score <6 (1^st^ quartile). The results of these analyses did not show a significant impact on the final ESs: OR = 3.71 [1.90–7.28] (*I* ^*2*^ = 86.71%, p<0.001) for compliance with 6-hour bundle (k = 8); OR = 1.83 [1.01–3.30] (*I* ^*2*^ = 66.14%, p = 0.019) for compliance to 24-hour bundle (k = 5); OR = 0.65 [0.53–0.80] (*I* ^*2*^ = 79.27%, p<0.001) for mortality (k = 16). The overall ES for mortality did not change when the analysis was restricted to studies for which the adjusted outcome data were available (OR = 0.67 [0.56–0.80], *I* ^*2*^ = 90.77%, p<0.001, k = 12).

## Discussion

The present systematic review and meta-analysis of 50 observational studies showed that the implementation of performance improvement programs increases compliance with the SSC guideline-based sepsis bundles and seems to be associated with decreased mortality in patients with sepsis, severe sepsis or septic shock.

Performance improvement initiatives varied from pure educational programs (including lectures, meetings, bedside training and educational materials) to interventions specifically aimed at inducing a variation in standard sepsis care (predefined order sets, clinical decision support and/or screening tools, activation of sepsis teams). Our analysis outlined the importance of medical/nurse staff education on sepsis pathogenesis, diagnosis and treatment. Education alone was able to improve compliance with the complete resuscitation and management bundles and was associated with a reduction in mortality. Pure process change programs were only able to improve compliance with the resuscitation bundle, but were still associated with a significant and consistent reduction in mortality. However, the largest increase in adherence to 6-hour and 24-hour bundles was induced by interventions including both an educational program and process changes, which were also associated with the greatest survival benefit. Therapies delivered early in the first 6 hours may have been the main determinant of survival in studies implementing process change interventions. A general increase in the attention to sepsis care may also have played a key role, rather than the implementation of the bundles *per se*.

In a prospective study by Levy et al [64], compliance to the complete sepsis resuscitation bundle was higher in the USA than in Europe; the unadjusted mortality was lower in the USA, but this difference disappeared after severity adjustment. In our analysis, the performance improvement programs induced a more than 8-fold increase in compliance with the complete 6-hour bundle in North-America. In Europe, compliance with 6-hour bundle increased by about 2 times. A higher percentage of North-American studies were performed in the ED (39% versus 23% in Europe), while in Europe the majority of studies were conducted in the ICU (61% versus 32% in North-America). It is reasonable that sepsis management in the ED mainly focus on earlier interventions. The interventions were associated with decreased mortality in North-America and non-significant survival benefit in Europe. Levy et al [64] showed that in the USA the majority of patients were admitted to the ICU from the ED, while in Europe 51.5% was admitted from the wards. Taken together, these data would suggest that an early more aggressive approach plays a key role in improving survival. Delay in sepsis diagnosis and/or admission to the ICU may be the major responsible for worse outcome, beyond the adherence to specific guideline-based targets.

In a retrospective study by Kang et al [65], care from experienced nurses (≥3 years of clinical experience), senior residents or board-certified emergency physicians was associated with higher compliance with the resuscitation bundle. We could not specifically evaluate the influence of healthcare staff experience on the effect of the quality improvement interventions. Interestingly however, the most consistent improvement in the adherence to the complete bundles was found among studies that reported the lowest initial compliance. In these cases, the performance improvement programs tended to be more strongly associated with a reduction in mortality. Efforts should be focused on the clinical settings where the adherence to SSC guidelines recommendations is poor, where quality improvement initiatives may be particularly beneficial in improving sepsis standard of care and patient outcome.

The implementation of performance improvement programs was more strongly associated with increased compliance with the resuscitation bundle when more severe patients were considered (severe sepsis and/or septic shock, higher severity score). Studies including only patients with septic shock showed the greatest survival benefit. It is reasonable that baseline compliance to the complete resuscitation bundle was lower for more severe patients, where physicians used to give priority to certain interventions over others. Our results would suggest that implementing a protocolized sepsis care may especially favor the prompt delivery of all recommended interventions in patients with higher risk of death.

In all the included studies sepsis treatment was based on 2004 or 2008 SSC-guidelines. In the updated 2012 SSC-guidelines [3], the bundles were revised: the resuscitation bundle was broken into two parts (3-hour and 6-hour), the management bundle was dropped. The guideline recommendations have undergone numerous changes as new more robust data emerged regarding type of fluids of choice, glucose control, corticosteroid administration [3]. Recombinant activated protein C was withdrawn from the market as it showed no consistent survival benefit [66]. Although this may limit the direct applicability of our results to the current clinical practice, it does not question the efficacy of performance improvement initiatives in promoting the best quality of care and increasing the clinicians’ awareness towards sepsis disease. In the present study, we showed that performance improvement initiatives increased the number of patients in whom the CVP and ScvO_2_ targets were achieved. Two recent multi-center RCTs [67, 68] failed to demonstrate a survival benefit of an early-goal directed therapy protocol based on CVP and ScvO_2_ measurement; protocolized care did not show any superiority over usual care, thus questioning the real advantages of SSC sepsis bundles. We did not evaluate the association between compliance with combined/individual bundle targets and mortality. Our data suggest a decrease in mortality following the implementation of performance improvement programs but cannot support any causality with the increased adherence to SSC guidelines. Observational studies are less methodologically rigorous than RCTs and prone to numerous biases. Secular trends may have been responsible for the observed decrease in mortality irrespectively of the interventions implemented. Of note, time-series analyses that are at lower risk for confounding by secular trends showed less extreme ES as compared to historically controlled or before-after investigations. Despite the failure of numerous RCTs in the past decade, recent reports suggest that the outcome of septic patients is improving over time [69, 70]. Judicious use of the SSC recommendations in the real clinical practice, in respect of the individual patient characteristics, may partly explain the discrepancy between these reports and data from RCTs. The worldwide implementation of performance improvement programs may have contributed substantially in reducing mortality from sepsis in the past decade, by increasing the attention of clinicians to sepsis care. These programs may be regarded as a tool for the divulgation of new robust evidences and their prompt implementation in the clinical practice. Levy et al. [38] reported a 7% decline in the risk of mortality for every additional quarter a site participates in the SSC program. We did not evaluate the relationship between time of participation in a performance improvement program and the compliance with SSC bundles achieved, nor did we consider the impact of time elapsed after the completion of the program, since only the final post-intervention phase was considered when compliance had been reported for different time periods. Nonetheless, it is worth noting that time-series analyses generally showed a persistent monotonic increase in compliance over time after the implementation of the interventions [8, 27, 38, 43, 46, 48, 54].

Our analysis has several limitations. Firstly, all the included studies were observational investigations and cannot thus support any causality between the quality improvement programs, increase in bundle compliance and reduction in mortality. Secondly, changes in adherence to SSC-guidelines or outcome over time may have occurred independently of the program implemented and could have influenced the results. Similarly, differences in disease severity or other confounders between the intervention and control groups could have biased the analysis. Nonetheless, whenever possible, we extracted adjusted data for the calculation of the ES for mortality (available for 12 studies out of 48); risk of bias and study quality were assessed and their influence on the results was explored by sensitivity analyses. Thirdly, we found high inconsistency between study findings, which may limit the validity of our data synthesis. We performed subgroup analyses in order to evaluate possible sources of heterogeneity, but cannot exclude the influence of unmeasured confounding factors. Of note, we could not evaluate the potential impact of any rewards or punishments delivered to healthcare providers based on the achieved compliance with the guidelines, as these were not generally reported in the studies. Fourth, although we performed an extensive review of the main electronic databases, we cannot be sure to have included all relevant studies. We did not consider studies that evaluated quality improvement initiatives implemented before the first publication of the SSC guidelines. In addition, it was not possible to take into account changes of the bundles occurring over time. Lastly, the possible publication bias may have influenced our results: the asymmetry of most of the funnel plots indicates that smaller studies tended to overestimate the association analyzed. We did not include unpublished studies, dissertations, or abstracts from conference proceedings. This may have contributed to the publication bias. However, we decided to consider only published materials in order to ensure that only higher quality, peer-reviewed studies were included in the meta-analysis. Similarly, the study selection was limited to English language studies because of cost reasons. This may have introduced an additional bias.

### Clinical implications

A widespread activation of educational programs that could increase the clinician’s awareness of the sepsis burden is desirable. These initiatives should not be restricted to the ICU setting but could be extended to the ED and the wards. These may include conference lectures, bedside teaching and simulation training focusing on the definition of sepsis syndrome and providing indications for an early diagnosis and prompt treatment. These programs should also be regarded as a tool for the divulgation of new evidences. Regular auditing on all patients treated in the ICU for severe sepsis or septic shock and feedback on the implementation of sepsis care are feasible initiatives that could prove useful in increasing the attention to the management of these patients. Additional simple tools, such as posters, bedside or pocket reminder cards, predefined order sets, flow charts and checklists may facilitate the early recognition of sepsis and implementation of the recommended therapies. A further step could be the introduction of electronic clinical decision support tools (e. g. sepsis alert systems) that may be developed in the future. Finally, it could be useful to have a “Sepsis Team” available in the hospital, to be activated as soon as possible in cases of suspected severe sepsis or septic shock, which may help to define the best diagnostic/therapeutic strategy.

## Conclusions

The present systematic review and meta-analysis on 50 observational studies showed that performance improvement programs were associated with increased compliance with sepsis resuscitation and management bundles and a reduction in mortality in patients with sepsis, severe sepsis or septic shock. Interventions including both an educational program and process changes were associated with the largest increase in compliance with SSC bundles and the greatest survival benefit. Studies including more severe patients showed a greater improvement in compliance with 6-hour bundle. Quality improvement initiatives represent a valuable tool to promote the best quality of care for septic patients.

## Key Messages

- Performance improvement programs were associated with an increase in compliance with the SSC-guideline based sepsis resuscitation and management bundles and in the adherence to individual bundle targets
- The implementation of quality improvement initiatives was associated with decreased mortality in patients with sepsis, severe sepsis or septic shock

## Supporting Information

S1 Fig Funnel plot and trim-and-fill analysis of studies that evaluated compliance with the complete 6-hour sepsis bundle. (PDF)

S2 Fig Funnel plot and trim-and-fill analysis of studies that evaluated compliance to individual 6-hour bundle targets. (A) Measure lactate; (B) Blood cultures; (C) Antibiotics; (D) Fluid resuscitation; (E) Measure central venous pressure; (F) Central venous pressure above 8 mmHg; (G) Measure SvO_2_; (H) SvO_2_ above 70%; (I) Mean arterial pressure above 65 mmHg; (L) Use of vasopressors.
(PDF)

S3 Fig Funnel plot and trim-and-fill analysis of studies that evaluated compliance to the complete 24-hour sepsis bundle. (PDF)

S4 Fig Funnel plot and trim-and-fill analysis of studies evaluating compliance with individual 24-hour bundle targets. (A) Lung protective ventilation; (B) Low-dose steroids; (C) Drotrecogin alfa (activated); (D) Glucose control.
(PDF)

S5 Fig Funnel plot and trim-and-fill analysis of studies that evaluated mortality. (PDF)

S1 Table Preferred Reporting Items for Systematic Reviews and Meta-Analyses (PRISMA) 2009 Check-list. (PDF)

S2 Table Study characteristics. (PDF)

S3 Table Outcomes measured in the analyzed studies. (PDF)

S4 Table Risk of bias and study quality assessment. (PDF)

S1 Text Search strategy used for PubMed (Medline) and adapted for the other electronic databases. (PDF)
