# Supplementary material for: Effect of Performance Improvement Programs on Compliance with Sepsis Bundles and Mortality: A Systematic Review and Meta-Analysis of Observational Studies
Source: PLoS One. 2015 May 6;10(5):e0125827. doi: 10.1371/journal.pone.0125827 (PMC4422717; doi:10.1371/journal.pone.0125827)

S1 Fig.

Funnel plot and trim-and-fill analysis of studies that evaluated compliance with the complete 6-hour sepsis bundle (k = 25). Open circles indicate the analyzed studies, full circles indicate the trimmed studies.

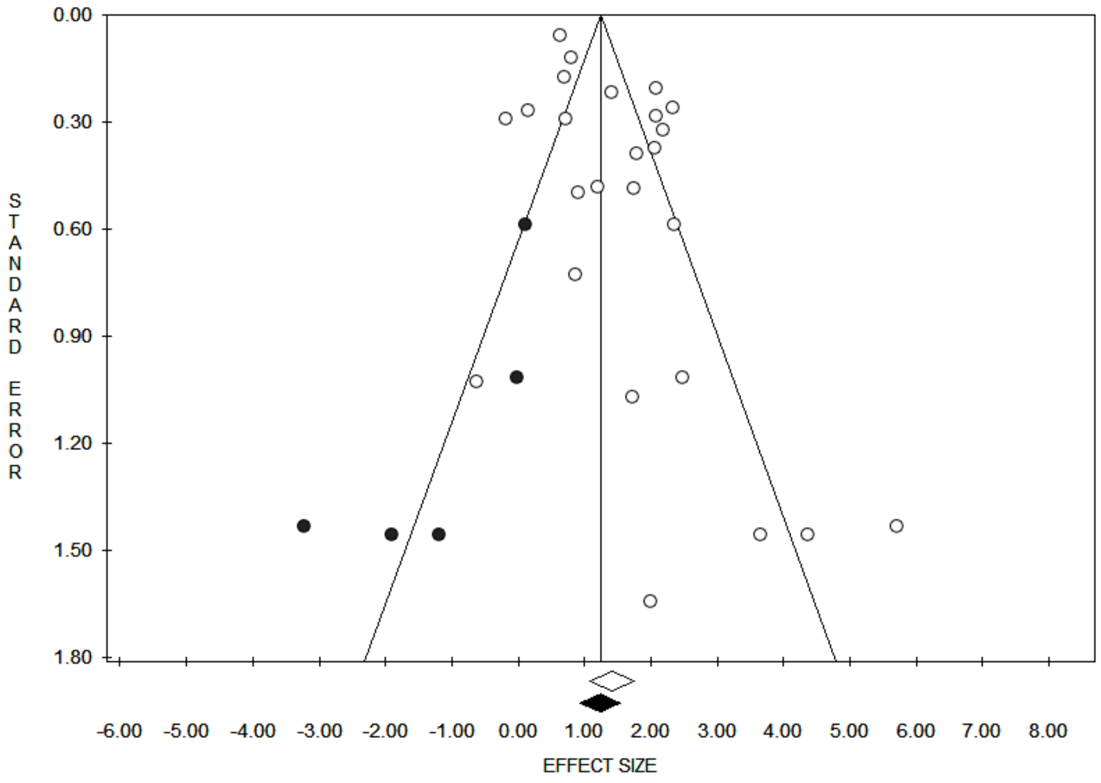

Supplement: S1 Fig — (PDF) [file pone.0125827.s001.pdf]
