# Supplementary material for: Effect of Performance Improvement Programs on Compliance with Sepsis Bundles and Mortality: A Systematic Review and Meta-Analysis of Observational Studies
Source: PLoS One. 2015 May 6;10(5):e0125827. doi: 10.1371/journal.pone.0125827 (PMC4422717; doi:10.1371/journal.pone.0125827)

S2 Fig.

Funnel plot and trim-and-fill analysis of studies that evaluated compliance to individual 6-hour bundle targets. Open circles indicate the analyzed studies, full circles indicate the trimmed studies.

(A) Measure Lactate. The trim-and-fill analysis did not show any obvious publication bias, however the Egger's linear regression test revealed an asymmetry of the funnel plot (t = 2.36, p=0.025)

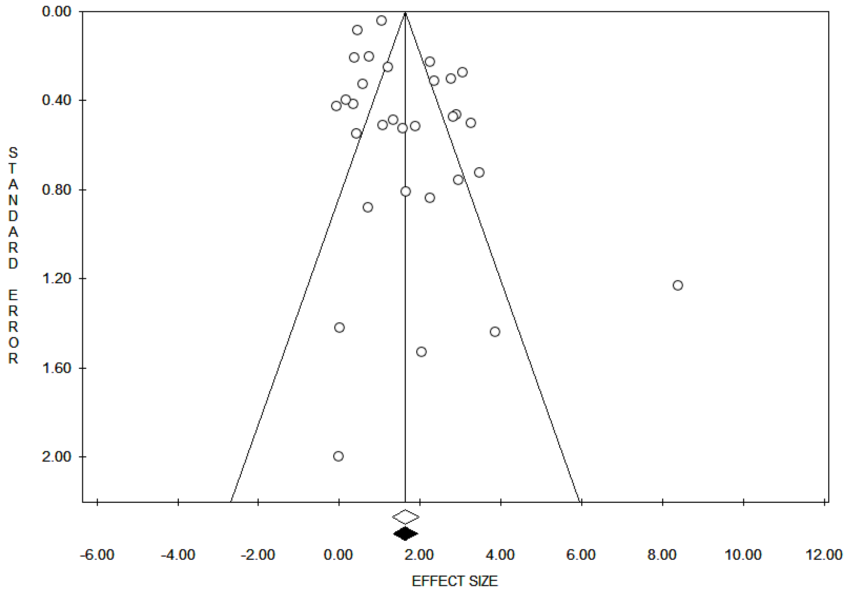

(B) Blood cultures. Trim-and-fill analysis and Egger's linear regression test did not show any asymmetry of the funnel plot.

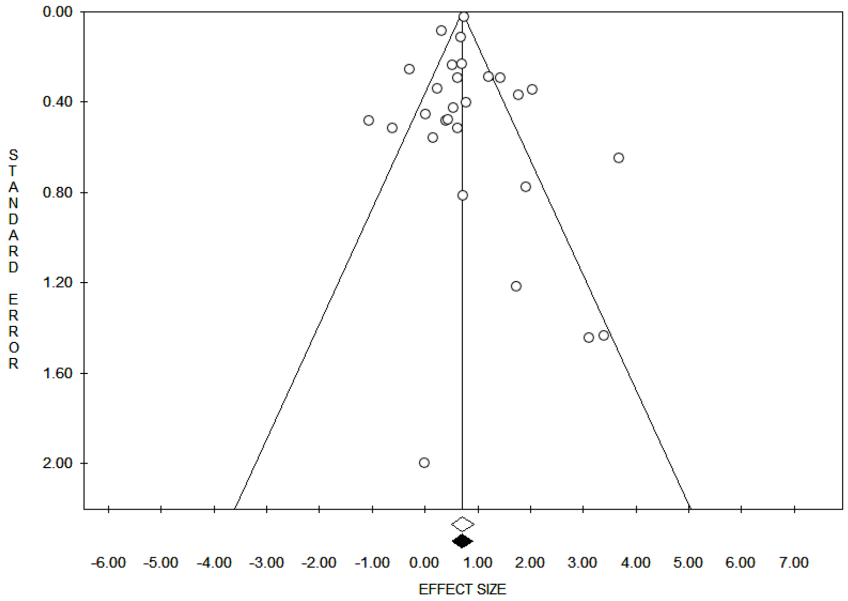

(C) Antibiotics. The trim-and-fill analysis revealed an asymmetry of the funnel plot (estimated ES = 2.06 [1.71-2.48] versus observed ES = 2.22 [1.86-2.66]; number of trimmed studies: 3). The Egger's linear regression test confirmed the possible presence of publication bias (t = 2.67, p = 0.012).

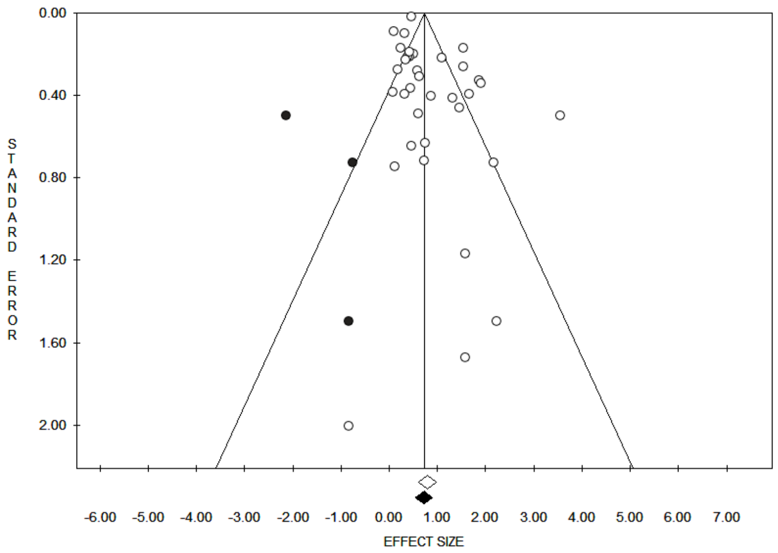

(D) Fluid resuscitation. The trim-and-fill analysis revealed an asymmetry of the funnel plot (estimated ES = 2.33 [1.62-3.35] versus observed ES = 3.22 [2.33-4.46]; number of trimmed studies: 5), although this was not confirmed by the Egger's linear regression test (t = 1.57, p = 0.130).

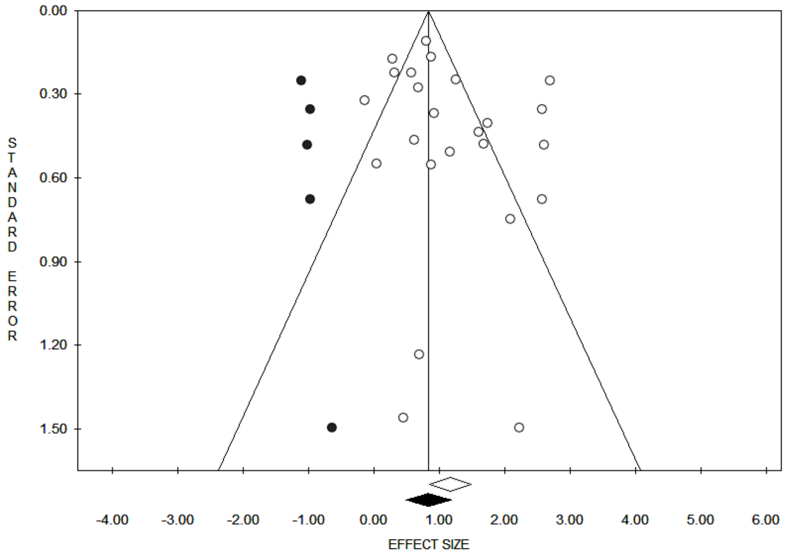

(E) Measure central venous pressure. The trim-and-fill analysis revealed an asymmetry of the funnel plot (estimated ES = 2.48 [1.31-4.68] versus observed ES = 2.77 [1.41-5.46]; number of trimmed studies: 1). The Egger's linear regression test confirmed the possible presence of a publication bias (t = 3.96, p = 0.007).

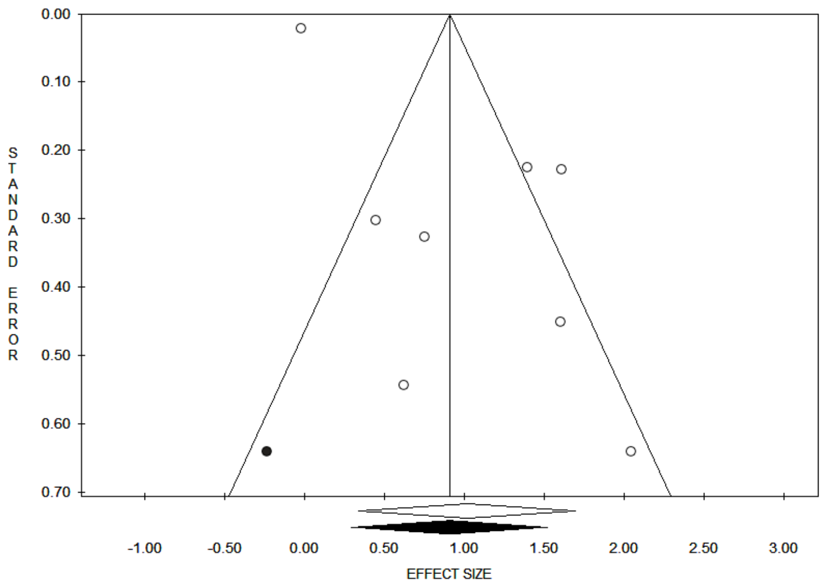

(F) Central venous pressure above 8 mmHg. The trim-and-fill analysis revealed an asymmetry of the funnel plot (estimated ES = 1.79 [1.37-2.33] versus observed ES = 1.91 [1.49-2.45]; number of trimmed studies: 2). The Egger's linear regression test showed a t = 1.99, p = 0.066.

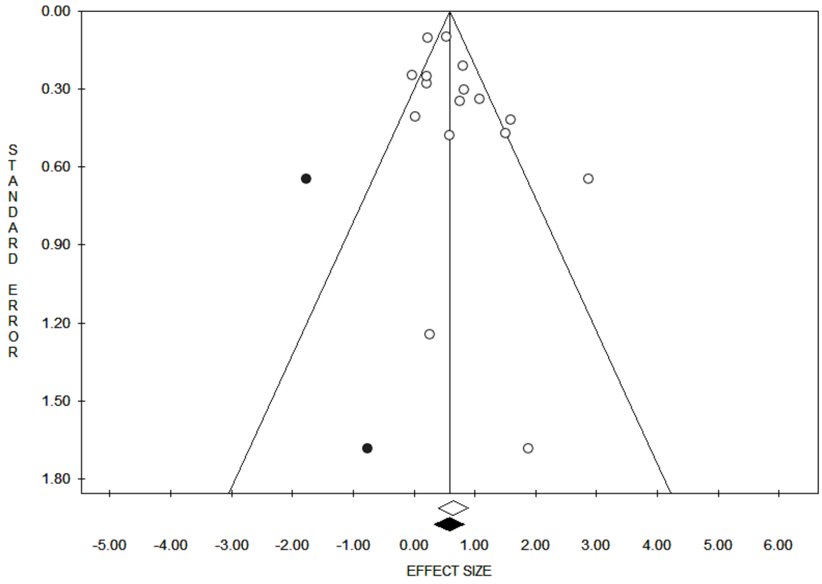

(G) Measure SvO_2. The trim-and-fill analysis revealed an asymmetry of the funnel plot (estimated ES = 6.33 [3.67-10.92] versus observed ES = 7.08 [4.25-11.80]; number of trimmed studies: 1), although the Egger's linear regression test did not show any obvious publication bias (t = 0.40, p = 0.701).

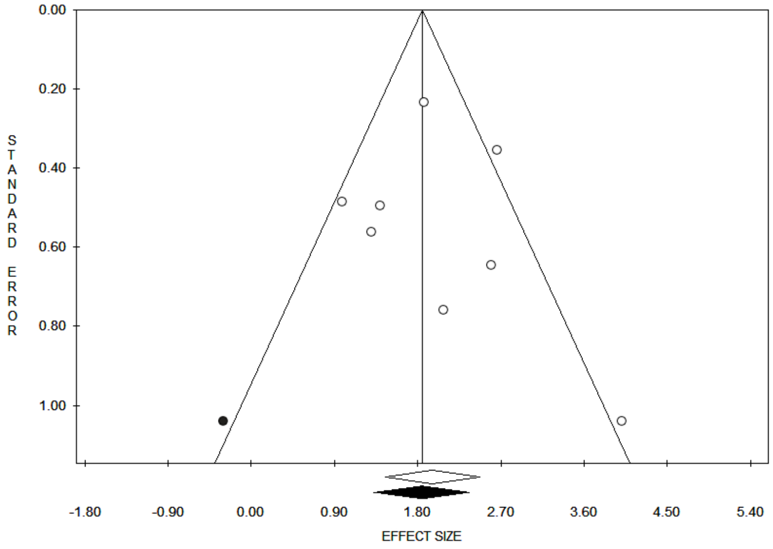

(H) SvO_2 above 70%. Trim-and-fill analysis and Egger's linear regression test (t = 1.90, p = 0.080) did not show any asymmetry of the funnel plot.

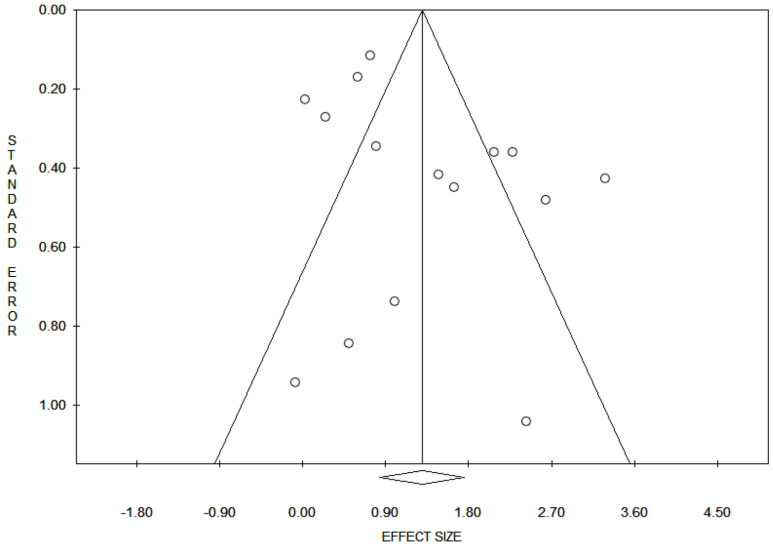

(I) Mean arterial pressure above 65 mmHg. The trim-and-fill analysis revealed an asymmetry of the funnel plot (estimated ES = 1.48 [0.98-2.25] versus observed ES = 1.55 [1.04-2.32]; number of trimmed studies: 1), although this was not confirmed by the Egger's linear regression test (t = 1.25, p = 0.252).

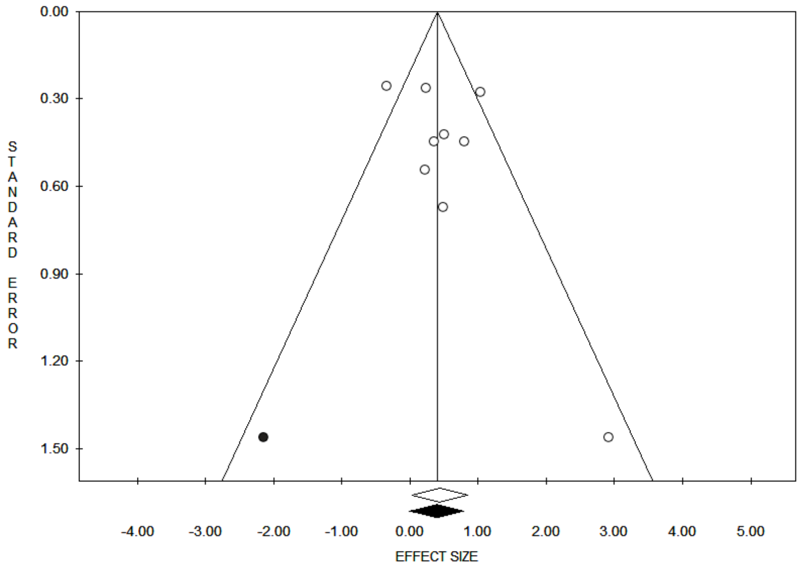

(L) Use of vasopressors. Trim-and-fill analysis and Egger's linear regression test (t = -1.42, p = 0.198) did not show any asymmetry of the funnel plot.

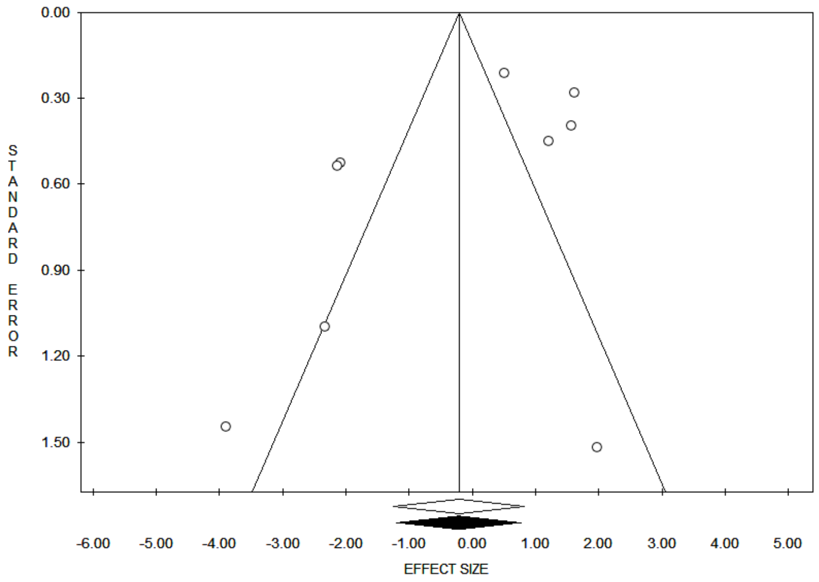

Supplement: S2 Fig — (A) Measure lactate; (B) Blood cultures; (C) Antibiotics; (D) Fluid resuscitation; (E) Measure central venous pressure; (F) Central venous pressure above 8 mmHg; (G) Measure SvO2; (H) SvO2 above 70%; (I) Mean arterial pressure above 65 mmHg; (L) Use of vasopressors. (PDF) [file pone.0125827.s002.pdf]
