# Supplementary material for: Effect of Performance Improvement Programs on Compliance with Sepsis Bundles and Mortality: A Systematic Review and Meta-Analysis of Observational Studies
Source: PLoS One. 2015 May 6;10(5):e0125827. doi: 10.1371/journal.pone.0125827 (PMC4422717; doi:10.1371/journal.pone.0125827)

S4 Fig.

Funnel plot and trim-and-fill analysis of studies evaluating compliance with individual 24-hour bundle targets. Open circles indicate the analyzed studies, full circles indicate the trimmed studies.

(A) Lung protective ventilation. The trim-and-fill analysis revealed an asymmetry of the funnel plot (estimated ES = 1.54 [1.00-2.37] versus observed ES = 1.63 [1.07-2.47]; number of trimmed studies: 1). The Egger's linear regression test showed a t = 2.19, p = 0.065.

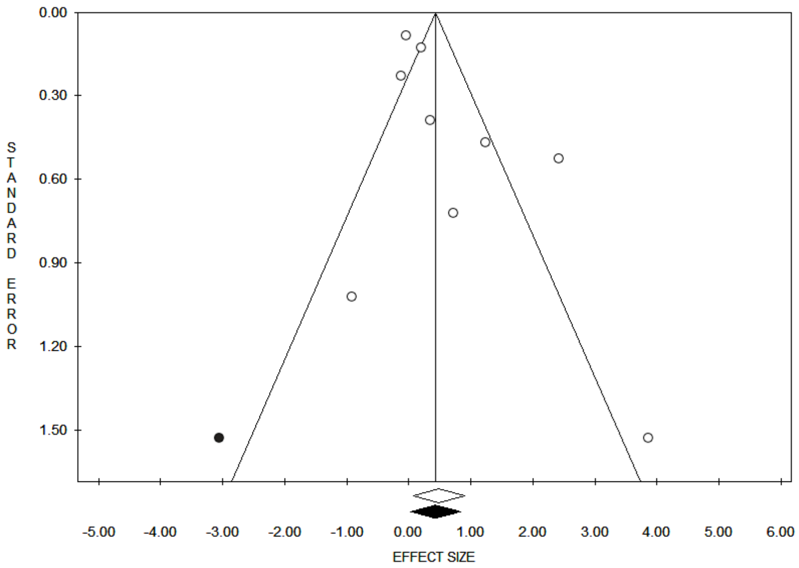

(B) Low-dose steroids. The trim-and-fill analysis revealed an asymmetry of the funnel plot (estimated ES = 1.48 [1.09-2.01] versus observed ES = 1.77 [1.37-2.29]; number of trimmed studies: 5). The Egger's linear regression test confirmed the possible presence of a publication bias (t = 2.66, p = 0.016).

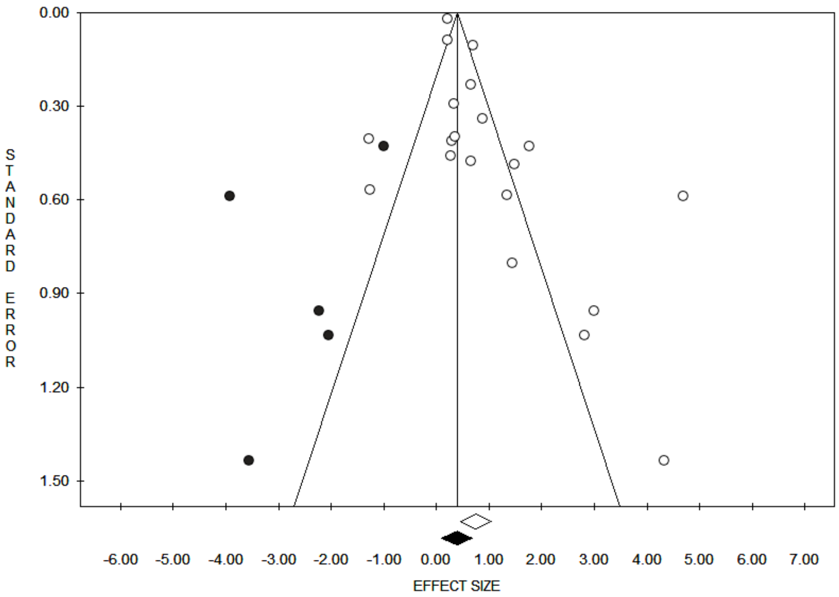

(C) Drotrecogin alfa (activated). Trim-and-fill analysis and Egger's linear regression test (t = 2.00, p = 0.066) did not show any asymmetry of the funnel plot.

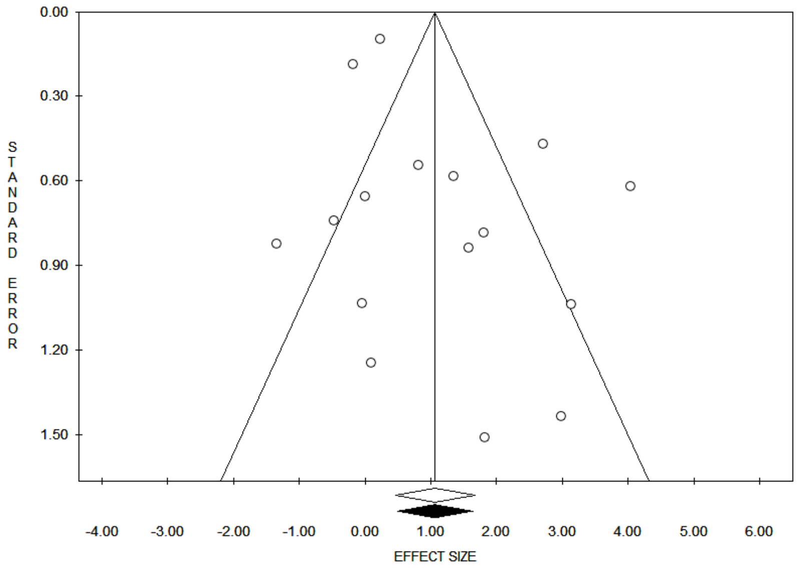

(D) Glucose control. Trim-and-fill analysis and Egger's linear regression test (t = 0.51, p = 0.621) did not show any asymmetry of the funnel plot.

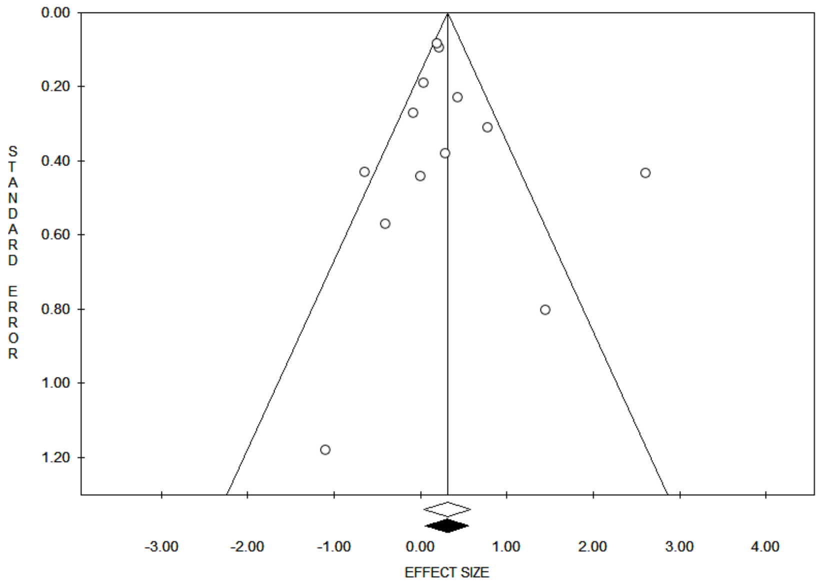

Supplement: S4 Fig — (A) Lung protective ventilation; (B) Low-dose steroids; (C) Drotrecogin alfa (activated); (D) Glucose control. (PDF) [file pone.0125827.s004.pdf]
