# Supplementary material for: Effect of Performance Improvement Programs on Compliance with Sepsis Bundles and Mortality: A Systematic Review and Meta-Analysis of Observational Studies
Source: PLoS One. 2015 May 6;10(5):e0125827. doi: 10.1371/journal.pone.0125827 (PMC4422717; doi:10.1371/journal.pone.0125827)

S5 Fig.

Funnel plot and trim-and-fill analysis of studies that evaluated mortality (k = 48). Open circles indicate the analyzed studies, full circles indicate the trimmed studies. The trim-and-fill analysis revealed an asymmetry of the funnel plot (estimated ES = 0.77 [0.71-0.83] versus observed ES = 0.66 [0.61-0.72]).

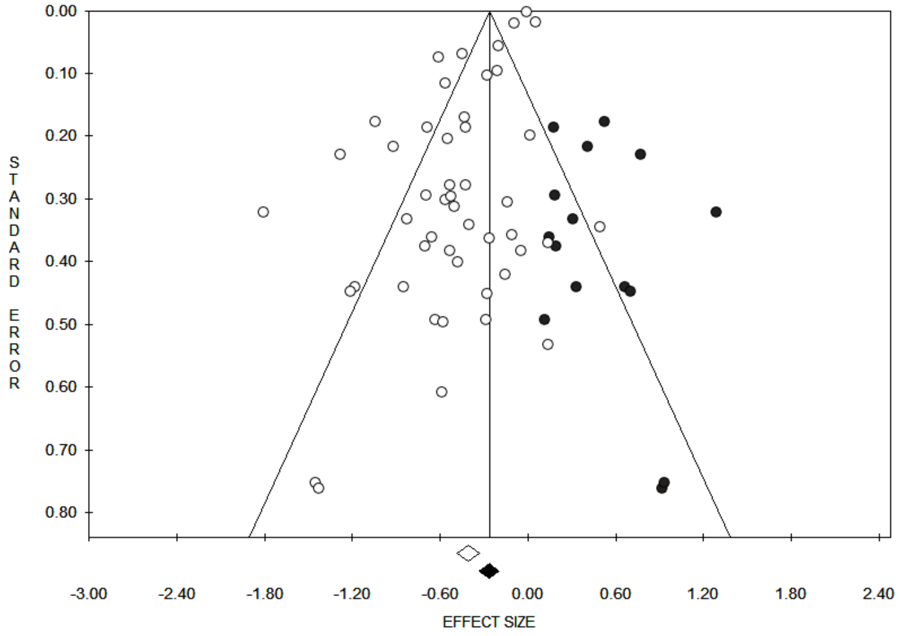

Supplement: S5 Fig — (PDF) [file pone.0125827.s005.pdf]
