# Supplementary material for: Effect of Performance Improvement Programs on Compliance with Sepsis Bundles and Mortality: A Systematic Review and Meta-Analysis of Observational Studies
Source: PLoS One. 2015 May 6;10(5):e0125827. doi: 10.1371/journal.pone.0125827 (PMC4422717; doi:10.1371/journal.pone.0125827)
Supplement: S2 Table — (PDF) [file pone.0125827.s007.pdf]

S2 Table

Study characteristics.

Study	Design	Country	Study period	Setting, teaching/non-teaching center	Sepsis severity	Admission	Performance improvement program	Type of program	Sample size, total (intervention/control)	Illness severity, mean (intervention/control)	Age, mean years (intervention/control)	H-mortality, mean % (intervention/control)
Berg 2013 [10]	retrospective cohort, single-center	USA	2004-2005	H, non-teaching	Severe sepsis + Septic shock	Mixed	Sepsis response team	PC	123 (36/87)	26.5 (30.8/24.7)	63 (57.1/65.4)	39.8 (25/43.7)
Bond 2013 [18]	retrospective case-control, single-center	Canada	2006-2008	ED, teaching	Severe sepsis + Septic shock	-	Electronic clinical practice guideline	PC	102 (51/51)	-	62 (62/62)	17 (17.7/15.7) *[a]*
Cannon 2012 [19]	historically controlled, multi-center	USA	-	H, mixed	Severe sepsis + Septic shock	Mixed	Sepsis alert, sepsis team, order sets, education, feedback	E + PC	5061 (4109/952)	-	-	31.6 (29/43)
Capuzzo 2012 [20]	prospective before-after, multi-center	Italy	2003-2007 (pre), 2009 (2nd period post)	H, mixed	Severe sepsis + Septic shock	Mixed	Courses and seminars, educational materials	E	333491 (276541/56950)	-	67.7 (67.6-68.5)	6.3 (6.6/6.2)
Castellanos-Ortega 2010 [21]	historically controlled, single-center	Spain	2004-2005 (pre), 2005-2008 (2nd period post)	ICU, teaching	Septic shock	Mixed	Lectures, training sessions, posters, pocket cards; "sepsis profile" for blood tests	E + PC	480 (384/96)	23.5 (23.2/24.6)	64 (64.5/62.2)	41.5 (37.5/57.3)
Chen 2013 [22]	retrospective before-after, multi-center	Taiwan	2000-2003 (pre), 2005-2008 (post)	H, mixed	Severe sepsis + Septic shock	Mixed	Educational program for intensivists	E	39760 (24858/14848)	-	65 (63.8/65.8)	46.8 (45.9/48.2) *[a]*
De Miguel-Yanes 2009 [23]	historically controlled, single-center	Spain	2005 (pre), 2007 (post)	ED, teaching	Sepsis + severe sepsis + septic shock	-	Training for residents, easy-to-read visual tools (flow-charts) for early diagnosis and treatment, creation of a High Dependency Unit in the ED	E + PC	103 (50/53)	17.2 (15.9/18.3)	71.3 (70.7/71.8)	20.4 (18/22.6)
El Solh 2008 [24]	historically controlled, single-center	USA	2001-2004 (pre), 2004-2007 (post)	H, teaching	Septic shock	-	Training for nurses, residents, physician assistants, sepsis protocol, preprinted order sets	E + PC	174 (87/87)	41 (42/40)	74.5 (74.6/74.5)	55.7 (54/57)
Ferrer 2008 [25]	prospective before-after, multi-center	Spain	2005 (pre), 2006 (post)	ICU, mixed	Severe sepsis + Septic shock	Mixed	Educational program and materials (posters, pocket cards) for physicians and nurses	E	2319 (1465/854)	21.2 (21/21.3)	62.2 (62.1/62.4)	41.2 (39.7/44)

Francis 2010 [26]	retrospective before-after, multi-center	Canada	2004 (pre), 2006 (post)	ED, teaching	Severe sepsis	-	Screening tool for sepsis, preprinted order sets, educational sessions	E + PC	213 (128/85)	-	64.1 (62.6/66.4)	-
Girardis 2009 [27]	time-series analysis, single-center	Italy	2005 (1st semester), 2007 (5th semester)	ICU, teaching	Severe sepsis + Septic shock	Mixed	Lectures and training, posters, sepsis team	E + PC	33 (20/13)	9.9 (8.4/12.3) *[b]*	62.6 (61/65)	48.5 (35/69)
Giuliano 2011 [9]	prospective before-after, single-center	USA	2006-2008	ICU, teaching	Sepsis + severe sepsis + septic shock	Mixed	Introduction of a computer application to assist physicians in sepsis diagnosis and treatment	PC	135 (70/65)	21.9 (22.3/21.4)	68.4 (69.1/67.6)	32.3 (33/31)
Gurnani 2010 [28]	prospective before-after, single-center	USA	2006-2008	ICU, teaching	Septic shock	-	Implementation of a SSC-based protocol with educational sessions	E + PC	118 (54/64)	27.5 (27/28)	56.9 (52/61)	48.3 (38.9/56.5)
Heppner 2012 [29]	prospective before-after, single-center	Germany	2008-2009	ICU, non-teaching	Sepsis + severe sepsis + septic shock	Medical	Implementation of a standardized order set, education and training	E + PC	122 (61/61)	-	80 (79.7/80.3)	49.2 (46.9/53.3)
Hoo 2009 [30]	retrospective before-after, single-center	USA	2007-2008	H, non-teaching	Sepsis + severe sepsis + septic shock	-	Education, preprinted order sets, feedback	E + PC	675 (365/310)	-	-	22.8 (17.3/29.4)
Jacob 2012 [31]	prospective before-after, multi-center	Uganda	2006 (pre), 2008-2009 (post)	H, non-teaching	Severe sepsis + Septic shock	-	Early sepsis management provided by a study medical officer	PC	671 (426/245)	-	34 (34/34)	38 (34.2/44.5)
Jeon 2012 [32]	historically controlled, single-center	Korea	2007-2009	ED, teaching	Severe sepsis + Septic shock	-	Lectures, bedside teaching and simulations for emergency department fellows, residents and nurses	E	366 (203/163)	7 (7/7) *[b]*	62.6 (63/62)	14.7 (11.8/18.4)
Jones 2011 [33]	Prospective before-after, single-center	USA	2004-2005 (pre), 2005-2007 (post)	ED, teaching	Severe sepsis + septic shock	-	Education and implementation of an early goal-directed protocol	E + PC	285 (206/79)	6.4 (7/5) *[b]*	56.5 (56/58)	20.3 (18/27)
Kuan 2013 [34]	prospective cohort, single-center	Singapore	2008 (pre), 2009 (4th period post)	ED, teaching	Severe sepsis + Septic shock	-	Lectures and training, reminder cards, personal feedback	E	117 (-/-)	20 (-/-)	58 (-/-)	26.5 (-/-)
Laguna-Perez 2012 [35]	prospective before-after, single-center	Spain	2008-2010	ICU, teaching	Severe sepsis + Septic shock	Mixed	Education and training, posters	E	125 (41/84)	25.8 (24.9/26.3)	64.2 (63.3/64.7)	39.2 (31.7/42.9) *[c]*

LaRosa 2012 [36]	prospective cohort, single-center	USA	2009	ICU, teaching	Severe sepsis + Septic shock	Mixed	Lectures and bedside teaching, screening tool, Code SMART (Sepsis Management Alert Response System), standardized order sets	E + PC	58 (34/24)	-	66.3 (70/61)	17.2 (9/29) *[d]*
Lefrant 2010 [37]	historically controlled, multi-center	France	2006	ICU, mixed	Severe sepsis + Septic shock	-	Educational program, posters, pocket cards, slide presentations	E	445 (215/230)	7.5 (8/7) *[b]*	64.5 (64/65)	33.7 (27/40) *[c]*
Levy 2010 [8]	time-series analysis, multi-center	USA, Europe, South America	2005-2008	H, mixed	Severe sepsis + Septic shock	Mixed	Educational materials, manual, meetings, cards, posters	E	3300 (509/2791)	-	-	33.8 (30.8/37)
Levy 2014 [38]	Time-series analysis, multi-center	USA, Europe, South America	2005-2012	H, mixed	Severe sepsis + septic shock	Mixed	Educational materials, manual, meetings, cards, posters	E	29470 (-/-)	-	-	32.8 (25.6/36.6)
MacRedmond 2010 [39]	historically controlled, single-center	Canada	-	ED, teaching	Severe sepsis + Septic shock	Mixed	Staff education (screening), sepsis algorithm, computerized physician order entry set, sepsis kit (antibiotics ready available at the ED), invasive hemodynamic monitoring made ready available at 2 high-intensity ED areas	E + PC	74 (37/37)	23.7 (22.6/24.9)	48.8 (45.1/52.6)	39.2 (27/51.4)
McKinley 2011 [40]	prospective before-after, single-center	USA	2008-2009	ICU, teaching	Severe sepsis + Septic shock	Surgical	Implementation of a computer protocol (open-loop system)	PC	206 (104/102)	27.5 (27/28)	58.5 (59/58)	19.4 (14/24)
Memon 2012 [41]	historically controlled, single-center	Saudi Arabia	2008-2011	ICU, non-teaching	Severe sepsis + Septic shock	Mixed	Educational program, evidence based written sepsis pathway, antibiotic recommendations	E + PC	298 (199/99)	21.3 (21.2/21.6)	66.2 (65/68.6)	24.5 (21.1/31.3) *[e]*
Micek 2006 [42]	prospective before-after, single-center	USA	2004-2005	ED, teaching	Septic shock	-	Standardized hospital order set, education and training on sepsis	E + PC	120 (60/60)	22.5 (23.3/21.7)	64.7 (61.4/68)	39.2 (30/48.3) *[c]*
Miller 2013 [43]	retrospective cohort, multi-center	USA	2004 (pre), 2010 (final period post)	ICU, mixed	Severe sepsis + Septic shock	Mixed	Large scale education about intent and elements of the bundle	E	1290 (965/325)	-	62.5 (-/-)	12.2 (8.7/21.2)
Moore 2009 [44]	retrospective before-after, single-center	USA	2006 (pre), 2007 (post)	ICU, teaching	Severe sepsis + Septic shock	Surgical	Sepsis screening tool	PC	136 (62/74)	-	-	30.1 (24.2/35.1)

Na 2012 [45]	prospective before-after, multi-center	Asia	2008-2009	ED, mixed	Severe sepsis + Septic shock	-	Lectures and medical simulations, bedside reminder cards, standardized bundle completion checklist, ICU-team (3 centers)	E + PC	183 (55/128)	22 (-/-)	63 (-/-)	29 (36.4/25.8)
Nguyen HB 2007 [46]	prospective before-after, single-center	USA	2003 (pre), 2005 (final period post)	ED, teaching	Severe sepsis + Septic shock	-	Lectures, bedside teaching, sepsis toolkit, pocket cards	E	78 (41/37)	32.4 (33.7/30.9)	66.4 (63.1/70.1)	-
Nguyen HM 2012 [47]	prospective before-after, single-center	USA	2003-2004 (pre), 2005-2006 (post)	ED, teaching	Severe sepsis + Septic shock	-	Lectures, reminders with SSC guidelines/sepsis bundle recommendations	E	96 (62/34)	28.6 (29/28)	71.2 (74/66)	37.5 (27.4/55.9)
Noritomi 2014 [48]	prospective before-after, multi-center	Brazil	2010 (pre), 2012 (final period post)	H, unclear	Severe sepsis + Septic shock	Mixed	Training program, screening tool (flow-chart), guidelines for antibiotic therapy, laboratory team, posters	E + PC	364 (161/203)	18.2 (16/20)	63.1 (62/64)	42 (25.5/55.2)
Palleschi 2013 [49]	retrospective before-after, multi-center	USA	2011 (pre), 2012 (post)	ED, teaching	Sepsis + severe sepsis + septic shock	-	Lectures, posters, badge extenders with guidelines for sepsis care	E	100 (53/47)	-	-	-
Patocka 2014 [50]	retrospective before-after, single-center	Canada	2005 (pre), 2008 (post)	ED, teaching	Severe sepsis + Septic shock	-	Training, triage screening tool	E + PC	355 (170/185)	-	73.9 (72.4/75.3)	18.6 (15.3/21.6)
Plambech 2012 [51]	prospective before-after, single-center	Denmark	2009-2010	ED, unclear	Sepsis + severe sepsis + septic shock	-	Electronically accessible guidelines, posters with diagnosis and treatment algorithms, pocket cards, checklists, training and support from key nurses and doctors	E + PC	75 (48/27)	-	-	-
Sawyer 2011 [52]	prospective case-control, single-center	USA	2008-2009	H, teaching	Sepsis + severe sepsis + septic shock	Medical	Computerized automatic real-time sepsis alert system	PC	270 (89/181)	17.6 (17.7/17.6)	52 (50.4/52.6)	11.2 (10.1/11.6)
Schramm 2011 [53]	prospective before-after, single-center	USA	(A) 2007 (pre), 2008 (post)<br>(B) 2007 (pre), 2009 (post)	ICU, teaching	Severe sepsis + Septic shock	Medical	(A) Daily auditing and weekly feedback<br>(B) Daily auditing and weekly feedback, sepsis team	(A) E<br>(B) E + PC	(A) 539 (272/267)<br>(B) 690 (423/267)	-<br>-	(A) 67.5 (68.7/66.3)<br>(B) 66 (65.8/66.3)	(A) 28.8 (28.7/30.3)<br>(B) 24.9 (22/30.3)
Seoane 2013 [54]	time-series analysis, single-center	USA	2008-2012	ICU, non-teaching	Severe sepsis + septic shock	Medical	Lectures, posters, pocket cards, online modules, order sets, feedback	E + PC	1105 (-/-)	Range 21-25 (23/21)	63 (-/-)	-(7.5/22)

Shapiro 2006 [55]	historically controlled, single-center	USA	2000-2001 (pre), 2003-2004 (post)	ED, teaching	Septic shock	Medical	Implementation of a sepsis protocol, training, lectures, cards, nursing flow sheets, computerized order sets	E + PC	130 (79/51)	24.1 (23.9/24.5)	69.6 (68/72)	25.3 (20.3/29.4)
Shiramizo 2011 [56]	prospective before-after, single-center	Brazil	2005-2006 (pre), 2009 (final period post)	ICU, non-teaching	Severe sepsis + Septic shock	Mixed	Lectures, e-learning, protocols, sepsis team	E + PC	217 (117/100)	23 (-/-)	66 (-/-)	33.6 (16.2/54)
Silverman 2011 [57]	(A) historically controlled, single-center	USA	(A) before 2006 (pre), 2006-2008 (post)	ICU, non-teaching	Severe sepsis + Septic shock	Surgical	(A) Introduction of a sepsis practice bundle by a sepsis team (education, support, order sets, surveillance)	E + PC	205 (186/19)	-	67.4 (67/72)	29.3 (28/42) *[d]*
	(B) prospective before-after		(B) 2006-2008 (pre), after Sept 2008 (post)				(B) Addition of a surgical intensivist and a surgical ICU care team	PC	254 (68/186)	-	66.2 (64/67)	26 (20/28) *[d]*
Sweet 2010 [58]	retrospective before-after, single-center	Canada	2004-2005 (pre), 2005-2006 (post)	ED, teaching	Sepsis + severe sepsis + septic shock	-	Development of a local sepsis protocol by a sepsis working group with preprinted order sets and a triage screening tool; education, posters with flow-charts	E + PC	59 (30/29)	24.4 (22/26.9)	58 (52.4/63.5)	25.4 (20/31)
Thiel 2009 [59]	retrospective before-after, single-center	USA	2003-2005 (pre), 2005-2006 (post)	H, teaching	Severe sepsis	Mixed	Hospital order sets based on SSC guidelines, education	E + PC	400 (200/200)	21 (20.2/21.8)	59.5 (60.5/58.5)	47.3 (39.5/55)
Tromp 2010 [60]	prospective before-after, single-center	Netherlands	(A) Jul 2006-Nov 2006 (pre), Nov 2006-Jun 2007 (post)	ED, teaching	Sepsis + severe sepsis + septic shock	-	(A) Development of a nurse-driven care bundle based sepsis protocol with screening and performance lists for nurses/physicians	(A) PC	(A) 606 (447/159)	-	(A) 59 (60/55)	(A) 6.1 (6/6.3)
			(B) Nov 2006-Jun 2007 (pre), Jun-Oct 2007 (post)				(B) Training on sepsis and performance feedback	(B) E	(B) 666 (219/447)	-	(B) 59.7 (59/60)	(B) 5.8 (5.5/6)
Vallée 2007 [61]	historically controlled, single-center	France	2005	ICU, teaching	Septic shock	-	Pentaxial target diagram (visual tool for hemodynamic parameters)	PC	80 (38/42)	62 (59.9/63.8) *[f]*	61 (63.7/58.4)	56.2 (52/59) *[c]*
Van Zanten 2014 [6]	Prospective time-series analysis, multi-center	Netherlands	2009-2013	ICU, mixed	Severe sepsis + Septic shock	Mixed	Educational materials, training, meetings, feedback	E	8387 (-/-)	83 (-/-) *[g]*	66.7 (-/-)	- (25.3/31.1)

Wang 2013 [62]	prospective before-after, single-center	China	2008 (pre), 2009 (post)	ED, teaching	Severe sepsis + Septic shock	-	Flow-chart, treatment algorithms, screening tool	PC	195 (117/78)	19.6 (19.5/19.7)	68.3 (69.9/65.9)	36.9 (31.6/44.8)
Westphal 2011 [63]	prospective before-after, multi-center	Brazil	Aug 2005-Oct 2006 (pre), Nov 2006-Nov 2007 (post)	H, non-teaching	Sepsis + severe sepsis + septic shock	Mixed	Screening protocol	PC	217 (115/102)	21 (21/22)	55.3 (55.4/55.2)	48.4 (36.5/61.7)

Illness severity is expressed as APACHE II score unless otherwise indicated. *ED* emergency department; *H* hospital; *ICU* intensive care unit; *E* education; *PC* process change; *[a]* 30-day mortality; *[b]* SOFA score; *[c]* 28-day mortality; *[d]* ICU-mortality; *[e]* 30-day hospital mortality; *[f]* SAPS; *[g]* APACHE IV
