# Supplementary material for: Effect of Performance Improvement Programs on Compliance with Sepsis Bundles and Mortality: A Systematic Review and Meta-Analysis of Observational Studies
Source: PLoS One. 2015 May 6;10(5):e0125827. doi: 10.1371/journal.pone.0125827 (PMC4422717; doi:10.1371/journal.pone.0125827)
Supplement: S3 Table — (PDF) [file pone.0125827.s008.pdf]

S3 Table

Outcomes measured in the analyzed studies.

Study	Compliance																Mortality
	6h-bundle	24h-bundle	Measure lactate	Blood cultures	Antibiotics	Fluid resuscitation	Measure CVP	CVP >8 mmHg	Measure SvO2	SvO2 >70%	MAP >65 mmHg	Vasopressors	Ventilation	Steroids	Glucose control	APC	
Berg 2013 [10]	*	*	*	*	*		*	*	*	*	*		*	*	*	*	*
Bond 2013 [18]			*	*	*	*	*		*			*		*			*
Cannon 2012 [19]																	*
Capuzzo 2012 [20]																	*
Castellanos-Ortega 2010 [21]	*	*	*	*	*	*		*		*	*		*	*	*	*	*
Chen 2013 [22]			*	*	*		*							*		*	*
De Miguel-Yanes 2009 [23]	*		*	*	*												*
El Solh 2008 [24]			*		*									*	*	*	*
Ferrer 2008 [25]	*	*	*	*	*			*		*			*	*	*	*	*
Francis 2010 [26]					*												
Girardis 2009 [27]	*	*		*	*	*				*			*	*	*	*	*
Giuliano 2011 [9]	*	*															*
Gurnani 2010 [28]						*											*
Heppner 2012 [29]			*	*	*	*			*								*
Hoo 2009 [30]																	*
Jacob 2012 [31]					*	*											*
Jeon 2012 [32]	*				*	*	*	*	*	*	*	*					*
Jones 2011 [33]												*				*	*
Kuan 2013 [34]	*		*	*	*	*		*		*	*						
Laguna-Perez 2012 [35]			*	*	*	*		*		*	*		*	*	*	*	*
LaRosa 2012 [36]			*	*	*	*	*	*	*	*					*	*	*
Lefrant 2010 [37]			*	*	*	*	*	*	*	*	*			*	*	*	*
Levy 2010 [8]	*	*	*	*	*	*		*		*			*	*	*	*	*
Levy 2014 [38]	*	*															*
MacRedmond 2010 [39]	*		*														*
McKinley 2011 [40]	*		*	*	*	*		*									*
Memon 2012 [41]	*		*	*	*	*		*		*	*						*

Micek 2006 [42]			*	*	*	*		*	*			*		*		*	*
Miller 2013 [43]																	*
Moore 2009 [44]																	*
Na 2012 [45]	*		*	*	*	*		*		*	*						*
Nguyen HB 2007 [46]	*		*		*	*								*			
Nguyen HM 2012 [47]			*	*	*	*								*	*	*	*
Noritomi 2014 [48]	*		*	*	*			*		*							*
Palleschi 2013 [49]			*	*													
Patocka 2014 [50]			*		*												*
Plambech 2012 [51]	*		*	*	*	*											
Sawyer 2011 [52]					*	*											*
Schramm 2011 [53]	*		*	*	*	*						*					*
Seoane 2013 [54]	*	*															*
Shapiro 2006 [55]					*							*		*			*
Shiramizo 2011 [56]	*	*	*	*	*			*		*			*	*	*	*	*
Silverman 2011 [57]																	*
Sweet 2010 [58]												*		*		*	*
Thiel 2009 [59]							*		*		*			*			*
Tromp 2010 [60]	*		*	*	*												*
Vallée 2007 [61]																	*
Van Zanten 2014 [6]	*	*	*	*	*	*	*					*	*	*	*	*	*
Wang 2013 [62]	*	*	*	*	*	*		*		*			*	*	*		*
Westphal 2011 [63]	*	*															*

*outcome measured; *CVP* central venous pressure; *APC* activated Protein C (Drotrecogin alfa activated)
