# Supplementary material for: Effect of Performance Improvement Programs on Compliance with Sepsis Bundles and Mortality: A Systematic Review and Meta-Analysis of Observational Studies
Source: PLoS One. 2015 May 6;10(5):e0125827. doi: 10.1371/journal.pone.0125827 (PMC4422717; doi:10.1371/journal.pone.0125827)
Supplement: S4 Table — (PDF) [file pone.0125827.s009.pdf]

S4 Table

Risk of bias and study quality assessment.

Study	Control for confounders		Newcastle-Ottawa Scale (cohort)									
	Severity of illness	Age	Representativeness of the exposed cohort	Selection of non-exposed cohort	Ascertainment of exposure	Demonstration that outcome was not present at start	Comparability of the two cohorts: severity of illness	Comparability of the two cohorts: age	Assessment of outcome	Follow-up long enough	Completeness of follow-up	Total NOS score
Berg 2013 [10]	no	no	*	*	*	*			*	*	*	7
Cannon 2012 [19]	no	no	*	*	*	*			*	*	*	7
Capuzzo 2012 [20]	no	adj (RR)	*	*	*	*		*	*	*		7
Castellanos-Ortega 2010 [21]	adj	adj	*	*	*	*	*	*	*	*	*	9
Chen 2013 [22]	no	no	*	*	*	*			*	*		6
De Miguel-Yanes 2009 [23]	no	no	*	*	*	*			*	*		6
El Solh 2008 [24]	bal	bal		*	*	*	*	*	*	*		7
Ferrer 2008 [25]	bal, adj	bal, adj	*	*	*	*	*	*	*	*	*	9
Francis 2010 [26]	no	bal	*	*	*	*		*	*	*		7
Girardis 2009 [27]	no	bal	*	*		*		*	*	*	*	7
Giuliano 2011 [9]	bal	bal	*	*	*	*	*	*	*	*	*	9
Gurnani 2010 [28]	bal	no	*	*	*	*	*		*	*		7
Heppner 2012 [29]	no	bal		*	*	*		*	*	*		6
Hoo 2009 [30]	no	no			*	*			*			3
Jacob 2012 [31]	no	adj (HR)		*	*	*		*	*	*	*	7
Jeon 2012 [32]	bal	bal	*	*	*	*	*	*	*	*	*	9
Jones 2011	no	bal	*	*	*	*		*	*	*		7

[33]												
Kuan 2013 [34]	no	no	*	*	*	*			*	*		6
Laguna-Perez 2012 [35]	bal	bal	*	*	*	*	*	*	*	*		7
LaRosa 2012 [36]	no	adj	*	*		*		*	*		*	6
Lefrant 2010 [37]	adj (HR)	bal	*	*	*	*	*	*	*	*		8
Levy 2010 [8]	no	no	*	*		*			*	*		5
Levy 2014 [38]	no	no	*	*		*			*	*		5
MacRedmond 2010 [39]	adj	no	*	*	*	*	*		*	*		7
McKinley 2011 [40]	bal	bal		*	*	*	*	*	*	*		7
Memon 2012 [41]	bal	bal	*	*	*	*	*	*	*	*	*	9
Micek 2006 [42]	adj	adj	*	*	*	*	*	*	*	*	*	9
Miller 2013 [43]	no	no	*	*	*	*			*	*		6
Moore 2009 [44]	no	no	*	*	*	*			*			5
Na 2012 [45]	no	no	*	*		*			*	*		5
Nguyen HB 2007 [46]	no	no	*	*	*	*			*	*		6
Nguyen HM 2012 [47]	bal	no	*	*	*	*	*		*	*		7
Noritomi 2014 [48]	no	bal	*	*	*	*		*	*	*		7
Palleschi 2013 [49]	no	no	*	*	*	*			*	*		6
Patocka 2014 [50]	no	bal	*	*	*	*		*	*	*		7
Plambech 2012 [51]	no	no	*	*	*	*			*	*		6
Schramm (A) 2011 [53]	adj	no		*	*	*	*		*	*	*	7
Schramm (B) 2011 [53]	adj	no		*	*	*	*		*	*	*	7
Seoane 2013 [54]	no	no			*	*			*	*		4

Shapiro 2006 [55]	bal	bal		*	*	*	*	*	*	*	*	8
Shiramizo 2011 [56]	no	no	*	*	*	*			*	*		6
Silverman (A) 2011 [57]	no	no			*	*			*			3
Silverman (B) 2011 [57]	no	no		*	*	*			*			4
Sweet 2010 [58]	no	no	*	*	*	*			*	*		6
Thiel 2009 [59]	adj	adj	*	*	*	*	*	*	*	*		8
Tromp (A) 2010 [60]	no	no	*	*	*	*			*	*		6
Tromp (B) 2010 [60]	no	no	*	*	*	*			*	*		6
Vallée 2007 [61]	bal	bal		*	*	*	*	*	*	*	*	8
Van Zanten 2014 [6]	adj	adj	*	*	*	*	*	*	*	*	*	9
Wang 2013 [62]	bal	bal	*	*	*	*	*	*	*	*		8
Westphal 2011 [63]	adj	bal	*	*	*	*	*	*	*	*		8

Newcastle-Ottawa Scale (case-control)

			**Case definition**	**Representativeness of cases**	**Selection of controls**	**Definition of controls**	**Comparability: severity of illness**	**Comparability: age**	**Ascertainment of exposure**	**Same method for ascertainment**	**Non-response rate (exclusions for missing data)**	**Total NOS score**
Bond 2013 [18]	no	match	*			*		*	*	*	*	6
Sawyer 2011 [52]	bal	bal	*	*		*	*	*	*	*		7

*Item fulfilled. The adjusted effect estimate calculated by the authors was odds ratio unless otherwise indicated in parentheses.

adj adjusted; *bal* balance; *match* matching; *RR* relative risk; *HR* hazard ratio.
