# Supplementary material for: Effect of Performance Improvement Programs on Compliance with Sepsis Bundles and Mortality: A Systematic Review and Meta-Analysis of Observational Studies
Source: PLoS One. 2015 May 6;10(5):e0125827. doi: 10.1371/journal.pone.0125827 (PMC4422717; doi:10.1371/journal.pone.0125827)
Supplement: S1 Text — (PDF) [file pone.0125827.s010.pdf]

Search strategy

Search strategy used for PubMed (Medline) and adapted for the other electronic databases.

1 - sepsis.ti,ab.

2 – septic shock.ti,ab.

3 – 1 or 2

4 – bundle.ti,ab.

5 – bundled care.ti,ab.

6 – bundled treatment.ti,ab.

7 – 4 or 5 or 6

8 – guidelines.ti,ab.

9 – surviving sepsis campaign.ti,ab.

10 – 8 and 9

11 – 3 and 7 and 10

12 – implementation program.ti,ab.

13 – performance improvement.ti,ab.

14 – quality improvement.ti,ab.

15 – 12 or 13 or 14

16 – 3 and 7 and 15

17 – 11 and 15
